# The TLR10–Vitamin D Axis Facilitates Osteogenic Differentiation of Mesenchymal Stem Cells In Vitro

**DOI:** 10.3390/cells15080697

**Published:** 2026-04-15

**Authors:** Anna Stierschneider, Benjamin Neuditschko, Isabella Fischer, Esther Hellmann, Daniel Zimmermann, Katerina Prohaska, Lisa Milchram, Franz Herzog, Christoph Wiesner

**Affiliations:** 1 Institute Biotechnology, IMC Krems University of Applied Sciences, 3500 Krems, Austria; anna.stierschneider@imc.ac.at (A.S.); isabella.fischer@imc.ac.at (I.F.); esther.hellmann@imc.ac.at (E.H.); daniel.zimmermann.dz@outlook.com (D.Z.); 2 Institute Krems Bioanalytics, IMC Krems University of Applied Sciences, 3500 Krems, Austria; benjamin.neuditschko@imc.ac.at (B.N.); lisa.milchram@imc.ac.at (L.M.); franz.herzog@imc.ac.at (F.H.); 3 Institute of Biotechnology & Analytics, Biotech Campus Tulln, University of Applied Sciences Wiener Neustadt, 3400 Tulln, Austria; katerina.prohaska@fhwn.ac.at

**Keywords:** toll-like receptor 10, mesenchymal stem cells, osteogenic differentiation, calcitriol

## Abstract

Bone regeneration requires tight coordination between mesenchymal stem cells (MSCs), immune signaling, and extracellular matrix remodeling. Yet, how atypical immune receptors contribute to this process remains unclear. Here, we identify Toll-like receptor 10 (TLR10) as a key regulator of osteogenic differentiation in human adipose-derived MSCs. Herein, ASC/TERT1 MSCs were engineered to overexpress or silence TLR10 using lentiviral vectors, and osteogenic differentiation (0–14 days) was assessed by metabolic assays—RT-qPCR of *COL1A2*, *ALPL* and *BGLAP*—Alizarin Red S staining, and quantitative mass spectrometry. Enhancing TLR10 expression promoted osteogenic gene programs, extracellular matrix organization, metabolic adaptation, and robust matrix mineralization, whereas TLR10 suppression maintained proliferative states and impaired osteoblast maturation. Proteomic analyses revealed that TLR10 selectively activates osteogenic, ECM-remodeling, and vitamin D-responsive pathways, while restraining programs antagonistic to differentiation. Notably, active vitamin D induced TLR10 expression and partially restored osteogenesis in TLR10-deficient cells, indicating that TLR10 is associated with vitamin D-driven bone formation. Together, beyond its established role in innate immunity, TLR10 emerges as a vitamin D-responsive regulator of mesenchymal stem cell osteogenesis, highlighting a potential therapeutic axis to enhance bone regeneration and osteogenic outcomes.

## 1. Introduction

**Figure 1:**
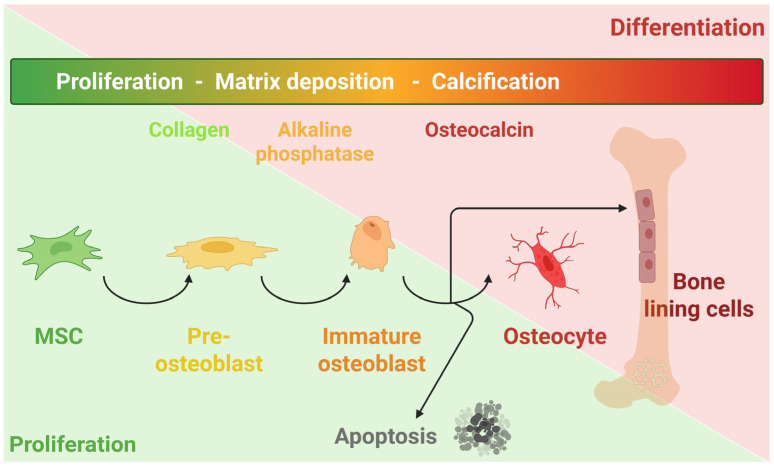
Schematic of MSC osteogenesis. MSCs actively proliferate during the early stages of osteogenesis. As they differentiate into osteoblasts, proliferation declines, and osteogenic markers are expressed, such as alkaline phosphatase during matrix maturation and osteocalcin during mineralization. Mature osteoblasts then become either bone-lining cells or osteocytes, or undergo apoptosis.

Bone regeneration is a complex and dynamic physiological process involving the activation of multiple cellular populations and molecular pathways that collectively restore the integrity and function of bone tissue [1]. Mesenchymal stem cells (MSCs) play a crucial role in this process due to their capacity for self-renewal and multilineage differentiation into osteoblasts [2], chondrocytes [3], adipocytes [4], myocytes [5], and neurons [6]. MSCs can be isolated from various sources, including bone marrow, adipose tissue, umbilical cord, placenta tissue, and dental pulp, and are key contributors in regenerative medicine because of their immunomodulatory and trophic properties that support angiogenesis and tissue repair [7,8]. During bone regeneration, MSCs serve as progenitors for osteoblasts and are recruited to resorption sites under the influence of signaling molecules such as transforming growth factor-β1 (TGF-β1). Once activated, these MSCs differentiate into pre-osteoblasts and begin synthesizing the unmineralized, collagen-rich matrix known as osteoid [9,10]. Subsequent deposition of calcium phosphate in the form of hydroxyapatite drives the mineralization of this matrix, ultimately leading to the formation of mature bone tissue. As bone formation proceeds, some osteoblasts become entrapped within the mineralized matrix and undergo terminal differentiation into osteocytes, which constitute 90–95% of all bone cells in adults [11]. These cells develop an extensive network of dendritic processes that extend through canaliculi, forming communication channels with neighboring osteocytes and surface-lining cells. Functionally, osteocytes act as the primary mechanosensors of the skeletal system, translating mechanical stimuli into biochemical signals that regulate both osteoblast and osteoclast activity, thereby maintaining bone homeostasis [12,13]. Covering the bone surface are bone-lining cells—flattened, quiescent descendants of osteoblasts—which were once thought to serve only in matrix maintenance but are now recognized as potential progenitors capable of reactivating and differentiating back into osteoblasts under anabolic or regenerative conditions. In contrast, osteoclasts, derived from the monocyte–macrophage lineage, are multinucleated cells responsible for bone resorption [14,15]. They degrade mineralized bone by secreting protons and proteolytic enzymes, such as cathepsin K, into an acidic microenvironment. Following resorption, these cells typically undergo apoptosis, thereby permitting the bone formation phase to begin again (Figure 1). The precise coordination of these cellular activities and the structural components of the extracellular matrix (ECM) enables the continuous remodeling of bone tissue to preserve mechanical integrity, adapt to mechanical stress, and maintain mineral homeostasis throughout life [16,17].

Among the key molecular regulators connecting immunity and bone biology are the Toll-like receptors (TLRs). These pattern recognition receptors (PRRs), expressed on both mesenchymal stem cells (MSCs) and various immune cells, recognize pathogen-associated molecular patterns (PAMPs) as well as damage-associated molecular patterns (DAMPs) [18]. TLR signaling affects MSC proliferation, migration, differentiation, and the production of inflammatory molecules through downstream cascades involving NF-κB, MAPK, and PI3K/Akt pathways [19,20,21]. TLRs influence MSC osteogenesis by altering the expression of osteogenic genes, regulating bone formation via cytokines, mediating MSC-immune cell interactions crucial for remodeling, and modulating extracellular vesicles (EVs) that affect or respond to TLR pathways [22,23,24].

Compared to human TLRs 1–9, the function of TLR10 in MSCs is poorly defined. Unlike other TLRs that activate canonical pro-inflammatory cascades via MyD88- or TRIF-dependent pathways, TLR10 attenuates NF-κB and MAPK activation and downregulates pro-inflammatory cytokine expression. It shares structural homology with TLR1 and TLR6, and can form heterodimers with TLR2, enabling selective ligand recognition while limiting pro-inflammatory secretion [25,26]. Therefore, elucidating the role of TLR10 in MSC osteogenesis is critical, as its immunomodulatory signaling may regulate NF-κB- and MAPK-mediated inflammatory inhibition of osteoblastic differentiation, providing a potential target to optimize MSC-driven bone regeneration through controlled immune–osteogenic crosstalk.

An additional level of regulation arises from the interaction between vitamin D signaling and TLR pathways. The active form of vitamin D, 1,25-dihydroxyvitamin D3 (calcitriol), promotes osteoblast differentiation through vitamin D receptor (VDR)-dependent transcriptional activation of osteogenic genes such as *COL1A1*, *ALPL*, and *BGLAP*, while simultaneously exerting anti-inflammatory effects [27,28,29]. Vitamin D has been shown to modulate TLR expression on immune cells, and upregulation of TLR10 by calcitriol has been documented in monocytes via VDR-RXRα interactions at the TLR10 promoter [30]. Furthermore, a study in human microglial cells demonstrated that calcitriol not only increased TLR10 mRNA and protein levels, but also promoted an anti-inflammatory, M2-polarized phenotype (characterized by upregulation of IL-10 and CCL17, and downregulation of IL-12 and TNF-α) under lipopolysaccharide stimulation [31]. These findings suggest a novel, vitamin D–TLR10 regulatory axis that may link immunomodulation and osteogenesis. More broadly, accumulating evidence indicates that nutritional and bioactive compounds, including vitamins, minerals, and phytochemicals, can modulate mesenchymal stem cell fate and function through interconnected metabolic and signaling pathways, further highlighting the relevance of vitamin D-mediated effects in MSC biology [32].

Taken together, these observations support a model in which MSCs, TLR10 signaling, and vitamin D pathways converge to regulate osteoblast differentiation and bone regeneration. Therefore, delineating this interplay is of great clinical relevance, as it may enable the development of targeted therapies that enhance MSC-based bone repair through selective modulation of the immune–osteogenic crosstalk.

## 2. Materials and Methods

### 2.1. Cells and Cell Culture

Human adipose-derived mesenchymal stem cells (ASC/TERT1) (female) were kindly provided by Evercyte (Vienna, Austria) and cultured in EGM-2 (Lonza, Basel, Switzerland), supplemented with G418 (InvivoGen, Toulouse, France). Growth medium for ASC/TERT1 with stable, integrated TLR10-expressing vector (TLR10-ASC/TERT1), TLR10-silencing vector (shTLR10-ASC/TERT1), or empty vector (mock-ASC/TERT1 or shCtrl-ASC/TERT1) was additionally supplemented with 1 µg/mL puromycin dihydrochloride (Thermo Fisher Scientific, Waltham, MA, USA). The cells were cultured and maintained in a humidified incubator at 37 °C with 5% CO_2_. Cells were subcultured or used for experiments at 80–90% confluency using 0.25% trypsin-EDTA (Thermo Fisher Scientific, Waltham, MA, USA). To induce osteogenic differentiation, ASC/TERT1 cells were seeded at a density of 3 × 10^5^ cells/2 mL growth medium in 6-well plates or at 1 × 10^4^ cells/100 µL growth medium in 96-well plates and allowed to adhere overnight. The following day, the growth medium was replaced with osteogenic induction medium (StemCell Technologies, Vancouver, BC, Canada) and cultured for 0–14 days, with medium changes every 3–4 days. The phenotype of ASC-TERT1 cells, including mesenchymal surface marker expression and multilineage differentiation capacity, has been previously characterized and shown to fulfill the established criteria for mesenchymal stromal cells [33]. TLR10 signaling was stimulated with 100 nM Calcitriol (MedChemExpress, Monmouth Junction, NJ, USA) for 24 h in growth medium or administered in conjunction with the osteogenic induction medium.

### 2.2. Engineering TLR10 Knockin and Knockdown Constructs

To explore the functional relevance of human TLR10, a TLR10-expressing vector (TLR10), a TLR10-silencing vector (shTLR10), and empty vectors (mock and shCtrl) were generated.

The cDNA of the open reading frame (ORF) of human TLR10 (Sino Biological, Beijing China) was subcloned into the lentiviral expression vector pEZ-Lv195 (GeneCopoeia, Rockville, MD, USA). For generation of the mock control construct, the TLR10-FLAG sequence was excised from the pCMV3-C-FLAG vector (Sino Biological) by PCR amplification using Phusion High-Fidelity DNA Polymerase and the following primers: Forward: 5′-P-TAATACGACTCACTATAGGG; Reverse: 5′-P-TGTGCGCACCGTAACATG. The lentiviral vector, pEZ-Z3377-Lv195 (GeneCopoeia), was linearized by PCR using the primers: Forward: CTGCGCAGAAAGGGCTCCTC; Reverse: AACTTGGACCTGGGAGTTGGG. Both PCR products were purified from agarose gels and ligated. The resulting constructs were transformed into *E. coli*, and plasmids were isolated from validated clones.

### 2.3. Transfection

For lentiviral production, the TLR10-expressing vector (TLR10-ASC/TERT1), TLR10-silencing vector (shTLR10-ASC/TERT1), and empty vectors (mock-ASC/TERT1 or shCtrl-ASC/TERT1) were individually transfected into ASC/TERT1 cells using the Lenti-Pac HIV Expression Packaging Kit (GeneCopoeia, Rockville, MD, USA), following the manufacturer’s instructions. Viral supernatants were collected 48 h post-transfection, filtered and either used immediately for cell transduction or stored at −80 °C for future use. For stable integration, ASC/TERT1 cells were infected with 0.5 mL of viral suspension that had been diluted in complete medium, which had been supplemented with 8 µg/mL polybrene (Merck, Darmstadt, Germany), in order to enhance transduction efficiency. After 24 h, the medium was replaced with fresh, complete growth medium. At 48 h post-infection, antibiotic selection was initiated using 1 µg/mL puromycin dihydrochloride (Thermo Fisher Scientific, Waltham, MA, USA). Following selection, individual single-cell clones were isolated, expanded, and validated for their expression profile.

### 2.4. RNA Extraction, cDNA Synthesis, and Quantitative Real-Time PCR

Total RNA was isolated and purified using the RNeasy Mini Kit (Qiagen, Hilden, Germany) according to the manufacturer’s protocol. Subsequently, 1 µg of total RNA was reverse-transcribed into complementary DNA (cDNA) using the High-Capacity cDNA Reverse Transcription Kit (Thermo Fisher Scientific, Waltham, MA, USA), following the manufacturer’s instructions. Quantitative real-time PCR (qPCR) was performed using pre-designed TaqMan Gene Expression Assays, each comprising a pair of unlabeled primers and a TaqMan probe labeled with a 5′-end FAM reporter dye, a minor groove binder (MGB), and a non-fluorescent quencher at the 3′-end. The following assays were used: TLR2 (Hs01872488_s1), TLR4 (Hs00152939_m1), TLR10 (Hs01935337_s1), COL1A2 (Hs01028956_m1), ALPL (Hs01029144_m1), BGLAP (Hs01587814_g1), and IPO8 (Hs00183533_m1, used as an endogenous control). Each qPCR reaction was prepared in a final volume of 20 µL, comprising 10 µL of TaqMan Gene Expression Master Mix (Thermo Fisher Scientific, Waltham, MA, USA), 1 µL of the respective TaqMan Gene Expression Assay, 5 µL of nuclease-free water (Ambion, Austin, TX, USA), and 4 µL of 1:10 diluted cDNA. Amplification reactions were performed using the QuantStudio 7 Flex Real-Time PCR System (Applied Biosystems, Waltham, MA, USA) with the following thermal cycling conditions: initial denaturation at 95 °C for 10 min, followed by 45 cycles of 95 °C for 20 s and 60 °C for 1 min. Fluorescence data were collected and analyzed using QuantStudio Real-Time PCR Software v1.3 (Applied Biosystems, Waltham, MA, USA). Relative mRNA expression levels of target genes were calculated using the comparative CT method (2^−ΔΔCT^), with IPO8 serving as the internal reference gene.

### 2.5. Protein Extraction and Western Blot Analysis

Cells were washed once with phosphate-buffered saline (PBS) and lysed in 100 µL of ice-cold lysis buffer containing 500 mM NaCl (Merck), 50 mM Tris-HCl (pH 7.4; Thermo Fisher Scientific, Waltham, MA, USA), 0.1% SDS (Carl Roth, Karlsruhe, Germany), 1% NP-40 (VWR), and 1 U of a protease and phosphatase inhibitor cocktail (Thermo Fisher Scientific, Waltham, MA, USA). Lysates were incubated on ice with gentle agitation for 20 min, followed by centrifugation at 12,000 rpm for 20 min at 4 °C. The resulting supernatants were collected, and total protein concentrations were quantified using the BCA Protein Assay Kit (Thermo Fisher Scientific, Waltham, MA, USA), following the manufacturer’s instructions. For protein denaturation, 1 µg of total protein was mixed with 4× Laemmli sample buffer (Bio-Rad, Hercules, CA, USA) containing 10% β-mercaptoethanol (Merck) and incubated at 70 °C for 10 min. Protein samples were separated by SDS-PAGE on 7.5% Mini-PROTEAN TGX Precast Protein Gels (Bio-Rad) at 100 V. Proteins were transferred onto nitrocellulose membranes (Bio-Rad) using the Trans Blot Turbo Transfer System with the standard semi-dry transfer program (35 min). Membranes were blocked overnight at 4 °C with 5% non-fat dry milk (New England Biolabs, Ipswich, MA, USA) diluted in PBS containing 0.05% Tween-20 to reduce non-specific binding. Membranes were subsequently incubated with the respective primary antibodies overnight at 4 °C. Bound antibodies were detected with an appropriate horseradish-peroxidase (HRP)-coupled secondary anti-rabbit (Cell Signaling, Danver, MA USA) incubated for 2 h at room temperature. Protein bands were visualized using the Clarity Western ECL Substrate (Bio-Rad), and chemiluminescence signals were detected using the ChemiDoc MP Imaging System (Bio-Rad, Hercules, CA, USA). The primary antibodies used in this study were as follows: anti-TLR2 (Thermo Fisher Scientific, Waltham, MA, USA), anti-TLR4 (US Biological, Salem, MA, USA), anti-TLR10 (US Biological), HRP-conjugated beta actin monoclonal antibody (Proteintech, Rosemont, IL, USA).

### 2.6. Cell Viability/Metabolism Assay

Cellular metabolic activity, as a proxy for viability and proliferation, was examined using PrestoBlue Cell Viability Reagent (Thermo Fisher Scientific, Waltham, MA, USA) and interpreted as an indirect indicator of proliferation and differentiation dynamics. Briefly, PrestoBlue was added directly to the culture medium in each well at a 1:10 dilution (*v*/*v*). Plates were incubated at 37 °C for 45 min in a humidified incubator. Fluorescence intensity, indicative of cellular metabolic activity, was measured using the SpectraMax i3x Multi-Mode Microplate Reader, equipped with a Transmitted Light (TL) detection cartridge (Molecular Devices, San Jose, CA, USA) at an excitation/emission wavelength of 555/585 nm.

### 2.7. Alizarin Red S Staining and Confocal Microscopy

Calcium deposition was assessed after 14 days of culture in osteogenic induction medium using Alizarin Red S staining. Briefly, cells were washed once with PBS (pH 7.4) and fixed with 10% formaldehyde (Thermo Fisher Scientific, Waltham, MA, USA) for 15 min at room temperature. After fixation, cells were rinsed with distilled water three times for 5 min and incubated with 2% (*w*/*v*) Alizarin Red S Solution (Merck) for 30 min at room temperature with gentle agitation. Excess dye was removed, and cells were washed thoroughly with distilled water until the background was clear. Stained calcium deposits, indicated by orange-red coloration, were imaged by confocal microscopy using the IXplore IX83 SpinSR Super Resolution Microscope System (Evident, Tokyo, Japan) and processed with the cellSens software v4.1 (Evident). For quantitative analysis of mineralization, entire wells of stained plates were scanned using the SpectraMax i3x Multi-Mode Microplate Reader equipped with a Transmitted Light (TL) detection cartridge (Molecular Devices), at an excitation/emission wavelength of 502/586 nm.

### 2.8. Quantitative Mass Spectrometry

#### 2.8.1. Sample Preparation

ASC/TERT1 cell lines with TLR10 knockin, TLR10 knockdown, and corresponding mock controls were used for proteomic analysis. Cells were cultured in standard growth medium (day 0) or osteogenic induction medium (StemCell Technologies, Vancouver, BC, Canada) for 7 or 14 days, with medium refreshed every 3–4 days. Cells were cultured in 6-well plates under standard conditions, and one day prior to harvesting, they were incubated in growth medium lacking fetal bovine serum to reduce serum protein contamination. At each time point, cells were detached using 0.25% trypsin–EDTA (Thermo Fisher Scientific), collected by centrifugation at 14,000× *g* for 10 min, and washed twice with phosphate-buffered saline (Thermo Fisher Scientific, Waltham, MA, USA) to remove residual media and serum proteins. Cell pellets were then resuspended in 80 µL lysis buffer consisting of 8 M urea (Merck, U5378-500G) and 50 mM ammonium bicarbonate (Merck, 09830-500G) to lyse the cells and denature the proteins. The whole cell lysate was transferred and centrifuged for 10 min at 14,000× *g* and stored at −20 °C until further processing.

After determination of the protein concentration using BCA assay (Sigma-Aldrich, St. Louise, MO, USA), 20 µg of protein was used for digestion. Samples were reduced and alkylated using TCEP (Sigma-Aldrich) and IAA (Sigma-Aldrich) and digested successively using Lys-C (FUJIFILM Wako Chemicals U.S.A. Corporation, Richmond, VA, USA) for one hour and Trypsin (Promega, Madison, WI, USA) for 16 h. Peptides were cleaned using Sep-Pak tC18 1 cc Vac Cartridges (Waters, Milford, MA, USA), dried, and stored at −20° until analysis occurred.

#### 2.8.2. HPLC-MS Analysis

Samples were analyzed using an Ultimate 3000 RSLCnano System coupled to an Orbitrap Eclipse Tribrid Mass Spectrometer (Thermo Fisher Scientific, Waltham, MA, USA).

The dried samples were dissolved in 20 µL Mobile Phase A (98% H_2_O, 2% ACN, 0.1% FA) and 2 µL were injected onto a PepMap RSLC EASY-Spray Column (C18, 2 µm, 100 Å, 75 µm × 50 cm, Thermo Fisher Scientific, Waltham, MA, USA). Separation occurred at 300 nL·min^−1^ with a flow gradient from 2 to 35% Mobile Phase B (2% H_2_O, 98% ACN, 0.1% FA) within 60 min, resulting in a total method time of 80 min. The mass spectrometer was operated in DIA mode with the FAIMS Pro System in positive ionization mode at CV-45. MS1 scans were acquired in a scan range of 350–1400 m·z^−1^ with a resolution of 120,000 @200 m·z^−1^. For DIA scans, the precursor mass range was set to 400–1000 m·z^−1^ with a 14 m·z^−1^ isolation window and 1 m·z^−1^ window overlap totaling in 43 independent scans. HCD fragmentation occurred at 30% NCE, and fragments were analyzed in the Orbitrap at a resolution of 30,000 @200 m·z^−1^.

To further deepen the analysis, a pool of all the samples was created and used for gas-phase fractionation (GPF) [34]. The sample pool was analyzed 6 times consecutively with smaller precursor mass ranges of 100 m·z^−1^ (400–500, 500–600, 600–700, 700–800, 800–900, 900–1000 m·z^−1^) and isolation windows of 4 m·z^−1^, with 2 m·z^−1^ window overlap.

For protein identification and quantification, Spectronaut (8.6.231227.55695) was used in direct DIA mode running the BGS Factory settings, using the human (Uniprot version 10.2021, 20,386 entries) protein database to predict the spectral library. GPF measurements were used as Library Extension Runs to maximize protein identification. Perseus (V2.0.6.0) [35] was used for statistical evaluation.

Differentially expressed proteins (DEPs) were analyzed using the NCBI DAVID (Database for Annotation, Visualization and Integrated Discovery) functional annotation platform. Functional enrichment analyses were conducted to identify significantly overrepresented Gene Ontology (GO) terms, including biological processes, molecular functions, and cellular components, as well as Kyoto Encyclopedia of Genes and Genomes (KEGG) pathways. Volcano plots depicting differential protein expression were generated in Perseus to visualize fold-change distributions and statistical significance. The resulting enrichment data were further evaluated and visualized in GraphPad Prism 9.3.0 (www.graphpad.com). Venn diagrams were constructed to illustrate shared and unique protein subsets specifically related to osteogenesis and vitamin D signaling, while network analyses were performed using KEGG pathway data to explore functional interconnections and potential mechanistic relationships between these signaling processes.

The mass spectrometry proteomics data have been deposited to the ProteomeXchange Consortium via the PRIDE [36] partner repository with the dataset identifier PXD071139.

### 2.9. Statistical Analysis

All experimental data represent the mean ± standard deviation of at least three technical replicates, with n denoting the number of independent biological replicates. Data analysis and graphical representation were performed using GraphPad Prism 9.3.0 (www.graphpad.com). Statistical significance was assessed by one-way or two-way ANOVA, followed by Dunnett’s or Šidák’s multiple comparisons tests, as indicated in the respective figure legends. A *p*-value of less than 0.05 (*p* < 0.05) was considered statistically significant.

## 3. Results

### 3.1. TLR10 Knockdown Attenuates, While TLR10 Knockin Augments, the Expression of Proteins Associated with Osteogenic and Vitamin D-Mediated Signaling Pathways in ASC/TERT1 Cells

**Figure 2:**
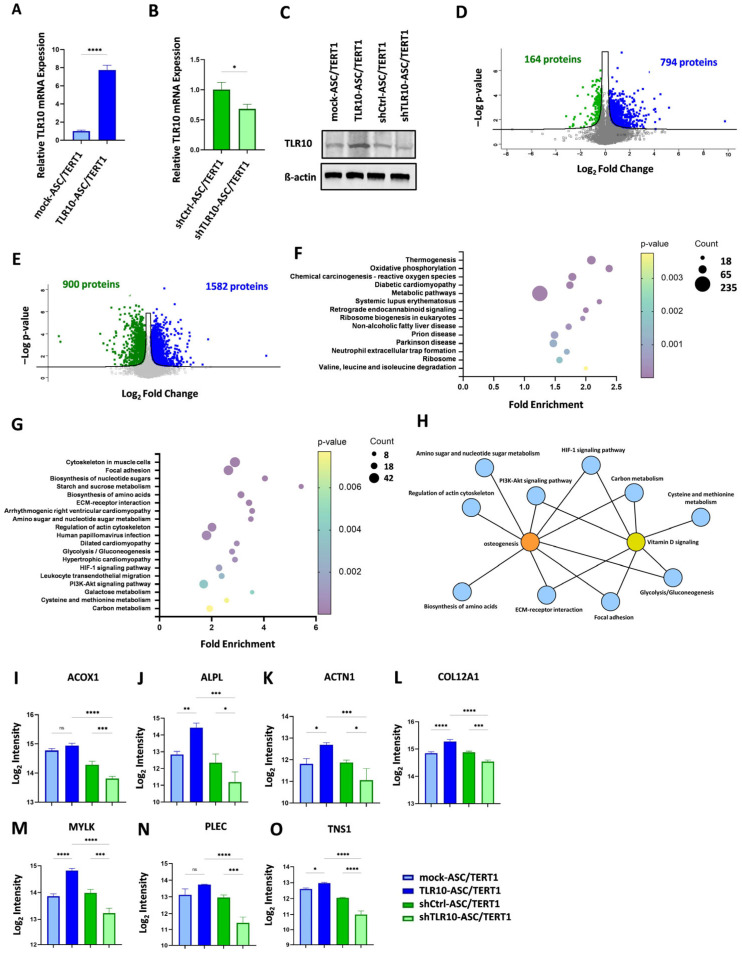
Characterization of the engineered TLR10 knockin (TLR10-ASC/TERT1) and TLR10 knockdown (shTLR10-ASC/TERT1) cell lines. (**A**,**B**) Quantitative PCR analysis of TLR10 mRNA and (**C**) Western blot analysis of TLR10 protein levels in mock, TLR10-overexpressing (TLR10-ASC/TERT1), and TLR10-silenced (shTLR10-ASC/TERT1) cells. (**A**,**B**) Data are shown as mean ± standard deviation (*n* = 3). Statistical analysis: Unpaired two-tailed Student’s *t*-test; * *p* < 0.05, **** *p* < 0.0001. (**C**) β-actin was used as a loading control. (**D**,**E**) Volcano plots display DEPs in TLR10-overexpressing (*n* = 3) (**D**) and TLR10-silenced (*n* = 3) (**E**) ASC/TERT1 cells, identifying significantly (*p*  <  0.05) upregulated (blue) and downregulated (green) proteins when compared to the corresponding mock control. The full set of proteins identified through our proteomic analysis and deposited in PRIDE can be found in the Supplementary Table S1. (**F**,**G**) Bubble plots of Kyoto Encyclopedia of Genes and Genomes (KEGG) pathway enrichment analysis, based on DAVID, of significantly (*p*  <  0.05) upregulated (**F**) and downregulated (**G**) proteins in TLR10-knockdown ASC/TERT1 cells versus mock control (*n* = 3). The size of the bubble indicates the number of DEPs involved, the color intensity (from violet to yellow) represents the *p*-value, and the *x*-axis displays the fold enrichment. (**H**) Network analysis of KEGG pathways enriched among downregulated proteins in TLR10-silenced ASC/TERT1 cells (*n* = 3). (**I**–**O**) Bar charts show log_2_ intensities of representative proteins significantly upregulated in TLR10-overexpressing cells and downregulated in TLR10-silenced cells, relative to the corresponding mock controls. Dashed and dotted lines represent the median and quartiles, respectively (*n* = 3). Statistical analysis: One-way ANOVA with Šidák’s post hoc test. ns = not significant, * *p* < 0.05, ** *p* < 0.01, *** *p* < 0.001, **** *p* < 0.0001.

To investigate the functional role of TLR10 signaling in MSCs, we generated stable TLR10 knockin and knockdown adipose-derived MSC lines (TLR10-ASC/TERT1 and shTLR10-ASC/TERT1), along with their respective mock controls (mock-ASC/TERT1 and shCtrl-ASC/TERT1). Across all conditions, cells display a characteristic, spindle-shaped, fibroblast-like morphology with elongated processes and growth in parallel arrays. No evident differences in cell morphology were detected between control, knockin, and knockdown conditions under standard culture conditions (Supplementary Figure S1). Stable modulation of TLR10 expression was confirmed by RT-qPCR and Western blot analysis. TLR10 mRNA levels increased nearly 8-fold in TLR10-ASC/TERT1 cells and were reduced to approximately 40–60% of control levels in shTLR10-ASC/TERT1 cells (Figure 2A,B). Consistent changes in TLR10 protein abundance were observed in the corresponding Western blot analysis (Figure 2C). Quantitative mass spectrometry was performed to characterize proteomic alterations associated with TLR10 expression in ASC/TERT1 cells. Comparison of TLR10-ASC/TERT1 cells with control cells identified 958 DEPs, including 794 upregulated and 164 downregulated proteins. In contrast, TLR10 knockdown (shTLR10-ASC/TERT1) cells exhibited a broader proteomic response, with 2482 DEPs, of which 1582 were upregulated and 900 were downregulated, relative to control. Pathway enrichment and network analyses revealed that osteogenic- and vitamin D-associated signaling pathways were positively enriched and interconnected in TLR10-overexpressing cells (Supplementary Figure S2), highlighting coordinated activation of pathways involved in osteoblast differentiation and mineralization. Conversely, these pathways were negatively enriched and displayed a disrupted network architecture in TLR10-silenced cells (Figure 2F–H), suggesting that TLR10 depletion impairs key regulatory interactions underlying osteogenic and vitamin D signaling. Further enrichment and Venn diagram analyses of cellular components, biological processes, and molecular functions supported these observations, demonstrating consistent positive enrichment of osteogenesis- and vitamin D-related terms in TLR10-ASC/TERT1 cells and negative enrichment in shTLR10-ASC/TERT1 cells (Supplementary Figures S3–S8). Bar charts (Figure 2I–O) illustrate the log_2_ intensities of representative proteins that were significantly upregulated in TLR10-overexpressing cells and downregulated in TLR10-silenced cells relative to mock controls. These data validate the upregulation of key proteins associated with metabolism (ACOX1), osteogenic differentiation (ALPL), extracellular matrix organization (ACTN1, COL12A1, PLEC, TNS1), and vitamin D-responsive signaling (MYLK) in TLR10 knockin cell lines, with corresponding downregulation observed in knockdown cells, in line with the enhanced osteogenic differentiation and mineralization observed in functional assay. Together, these findings indicate that TLR10 plays a substantial role in regulating osteoblast differentiation, potentially through vitamin D-responsive signaling pathways, and highlight its potential involvement in maintaining bone formation.

### 3.2. TLR10 Promotes Osteogenesis in ASC/TERT1 Cells by Regulating Cellular Metabolism/Proliferation, Differentiation, Extracellular Matrix Maturation, and Calcification

To assess the functional impact of TLR10 on osteogenic differentiation, we evaluated cell metabolism/proliferation using PrestoBlue assay, quantified the expression of osteogenic marker genes (*COL1A2*, *ALPL*, and *BGLAP*) by RT–qPCR, and analyzed matrix mineralization through Alizarin Red S staining.

**Figure 3:**
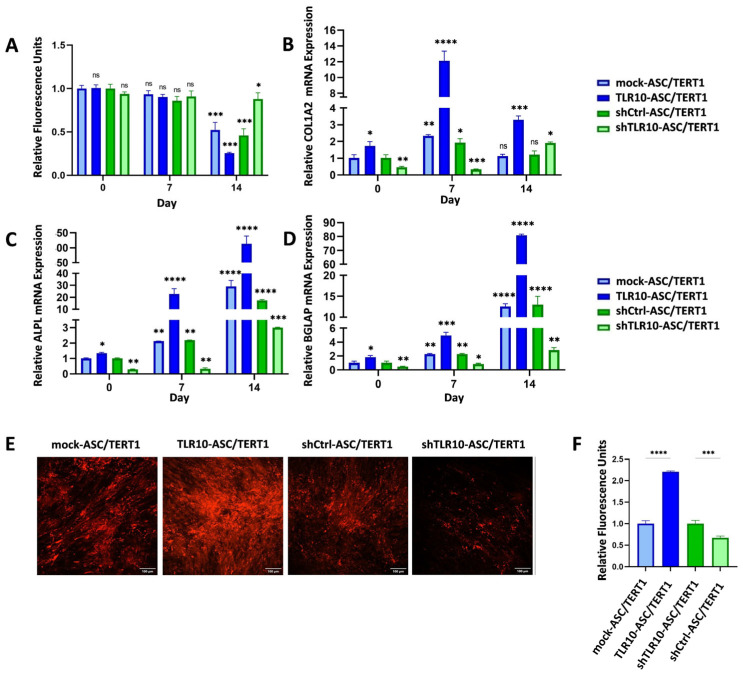
Analysis of cellular metabolism, osteogenic differentiation, extracellular matrix deposition, and mineralization in TLR10-modulated ASC/TERT1 cells. (**A**) Cellular metabolic activity, measured by PrestoBlue on days 0, 7, and 14. (**B**) Relative collagen mRNA expression levels. (**C**) Alkaline phosphatase (ALPL) mRNA expression. (**D**) Osteocalcin mRNA expression at days 0, 7, and 14 confirms enhanced late-stage osteogenic differentiation under TLR10 activation and impaired differentiation upon TLR10 silencing. (**A**–**D**) Data are shown as mean ± standard deviation (*n* = 3). Statistical analysis: Two-way ANOVA with Šidák’s post hoc test; ns = not significant, * *p* < 0.05, ** *p* < 0.01, *** *p* < 0.001, **** *p* < 0.0001. TLR10 knockin cells at days 0–7 were normalized to mock-ASC/TERT1 cells at day 0 and TLR10 knockdown cells at days 0–7 were normalized to shCtrl-ASC/TERT1 cells at day 0. (**E**,**F**) Extracellular matrix mineralization, assessed by Alizarin Red S staining at day 14, with representative confocal images and corresponding quantitative fluorescence well scans. TLR10 overexpression increased calcium deposition, whereas TLR10 knockdown reduced mineralization. (**E**) Scale bars = 100 µm. (**F**) Data are shown as mean ± standard deviation (*n* = 3). Statistical analysis: One-way ANOVA with Šidák’s post hoc test. *** *p* < 0.001, **** *p* < 0.0001.

Cellular metabolism/proliferation during osteogenic differentiation were assessed using the PrestoBlue assay in TLR10 knockin, TLR10 knockdown, and corresponding mock control ASC/TERT1 cells at days 0, 7, and 14 (Figure 3A). At the early time points (day 0 and day 7), all groups exhibited comparable metabolic activity, indicating similar proliferation rates prior to and during the initial phase of differentiation. As osteogenic differentiation progressed, metabolic activity declined in accordance with reduced proliferation and increased matrix maturation. By day 14, TLR10-ASC/TERT1 cells exhibited a remarkable decrease in PrestoBlue signal, retaining approximately 25% of the day 0 activity, consistent with terminal differentiation. Mock controls showed a solid decline to roughly 50%, reflecting normal differentiation-associated growth arrest. In contrast, shTLR10-ASC/TERT1 cells maintained about 90% of their initial metabolic activity, suggesting impaired progression toward osteogenic maturation (Figure 3A). These results indicate that TLR10 expression facilitates the metabolic shift associated with osteogenic differentiation, whereas TLR10 silencing preserves a proliferative phenotype, consistent with delayed or incomplete osteoblast differentiation.

To further evaluate osteogenic differentiation at the molecular level, the expression of key osteogenic markers was measured by RT–qPCR over a 14-day differentiation period in TLR10 knockin, TLR10 knockdown, and control ASC/TERT1 cells (Figure 3B–D). *COL1A2*, a marker of proliferation and early extracellular matrix formation, was strongly upregulated in TLR10-ASC/TERT1 cells on day 7, whereas its expression in shTLR10-ASC/TERT1 cells remained low and fell below that of control cells, indicating a delayed onset of matrix synthesis, supporting a role for TLR10 in regulating extracellular matrix production (Figure 3B). *ALPL*, indicative of matrix maturation, showed robust induction in TLR10 knockin cells on days 7 and 14 (Figure 3C). In contrast, mock controls increased moderately, while TLR10 knockdown cells exhibited only a minor upregulation that remained significantly below mock levels, indicating impaired matrix maturation. *BGLAP*, a late-stage marker of mineralization, was markedly elevated in TLR10-ASC/TERT1 cells on day 14, moderately induced in controls, and substantially reduced in shTLR10-ASC/TERT1 cells, reflecting deficient terminal differentiation (Figure 3D). These transcriptional profiles indicate reduced proliferation and enhanced mineral deposition in TLR10 knockin ASC/TERT1 cells, moderate differentiation in controls, and sustained proliferation with limited calcification in shTLR10-ASC/TERT1 cells.

Alizarin Red S staining was performed on day 14 to visualize calcium deposition in differentiated ASC/TERT1 cells. Both confocal microscopy and quantitative well-scan analysis (plate reader measurement of relative fluorescence units, RFU) were conducted to evaluate mineralized matrix formation across TLR10 knockin, TLR10 knockdown, and control cell lines. Confocal imaging revealed markedly increased calcium deposition in TLR10-ASC/TERT1 cells, with dense and extensive Alizarin Red S staining relative to control cultures, whereas shTLR10-ASC/TERT1 cells exhibited visibly reduced mineralization. Quantitative analysis confirmed these observations: RFU values in TLR10-ASC/TERT1 cells were more than 2-fold higher than controls, while shTLR10-ASC/TERT1 cells retained below 70% of control fluorescence intensity (Figure 3E,F). Collectively, these results demonstrate that TLR10 enhances osteogenic mineralization, while its depletion significantly attenuates calcium deposition during differentiation, reinforcing a functional role of TLR10 in promoting osteoblast maturation and matrix mineralization.

### 3.3. TLR10 Modulation Alters, Osteogenic, Proliferative, and Extracellular Matrix-Associated Proteomic Profiling in ASC/TERT1 Cells

**Figure 4:**
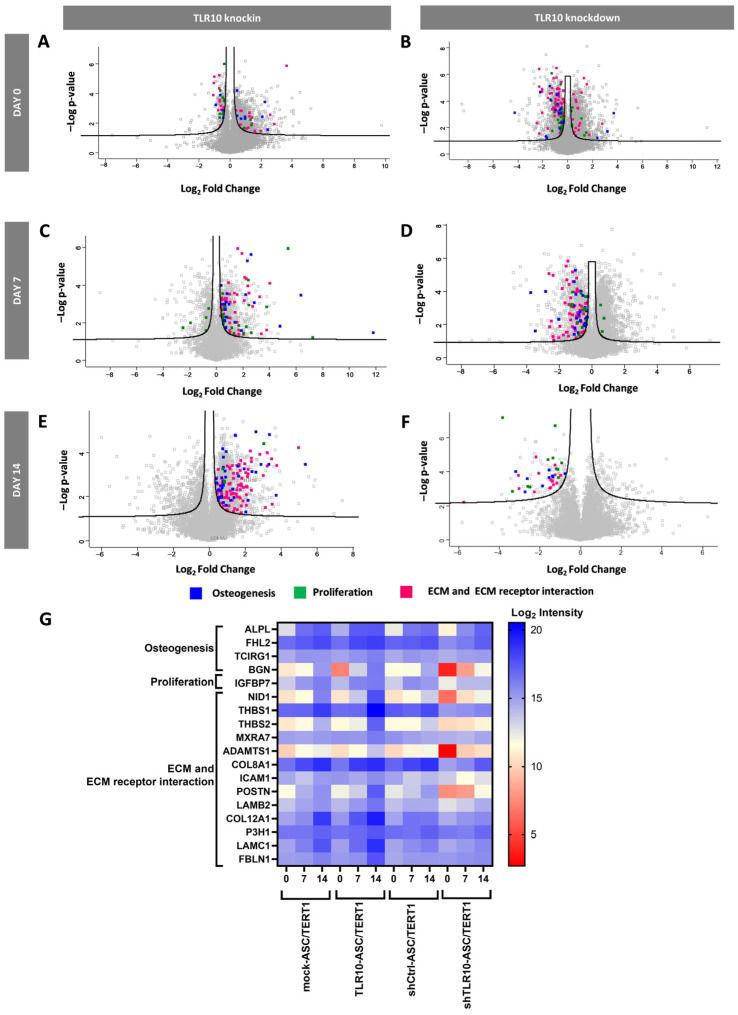
Proteomic profiling of differentially expressed proteins in TLR10-modulated ASC/TERT1 cells during osteogenesis. (**A**–**F**) Volcano plots depict proteomic alterations in TLR10-overexpressing (TLR10-ASC/TERT1) and TLR10-silenced (shTLR10-ASC/TERT1) cells at day 0, day 7, and day 14 of osteogenic induction. Quantitative, label-free proteomics (LFQ) identified significantly differentially expressed proteins (DEPs), with log_2_ fold changes in protein abundance plotted against statistical significance (−log_10_ *p*-value) (*n* = 3). Significantly upregulated proteins are displayed on the right, and downregulated proteins on the left. Black lines indicate thresholds for fold change and statistical significance. The full set of proteins identified through our proteomic analysis and deposited in PRIDE can be found in the Supplementary Table S1. (**G**) Heatmap showing the distribution of DEPs related to osteogenic differentiation, proliferation, and extracellular matrix formation in TLR10-overexpressing and TLR10-silenced ASC/TERT1 cells compared to the respective mock control at days 0, 7, and 14 of osteogenesis (*n* = 3). Protein abundance values are represented as log_2_ intensities, illustrating temporal and condition-dependent variations in protein expression.

Volcano plots illustrating proteomic changes in TLR10 knockin and TLR10 knockdown ASC/TERT1 cells compared to their respective mock controls at days 0, 7, and 14 of osteogenic differentiation are shown in Figure 4A–F. Each plot depicts the log_2_ fold change in protein abundance versus log *p*-value, with proteins associated with osteogenesis highlighted in blue, proliferation in green, and ECM or ECM–receptor interaction in pink. On day 0, prior to induction, TLR10-ASCs/TERT1 cells displayed a relatively balanced distribution of up- and downregulated proteins, with a few osteogenesis- and ECM-related proteins already modestly upregulated. This finding suggests that TLR10 overexpression may prime MSCs toward a more osteogenic and matrix-remodeling phenotype in MSCs, even before the onset of differentiation stimuli. In contrast, shTLR10-ASC/TERT1 cells exhibited a leftward shift, with downregulation of osteogenic and matrix-remodeling proteins, implying a more proliferative, undifferentiated basal state. By day 7, both cell lines showed distinct proteomic remodeling patterns. TLR10-ASC/TERT1 cells displayed a clear enrichment of osteogenesis-related proteins, appearing on the right side of the volcano plot, including several involved in ECM and ECM–receptor interactions, consistent with active matrix deposition and osteogenic progression. Conversely, shTLR10-ASC/TERT1 cells exhibited the opposite trend, suggesting delayed differentiation and sustained proliferative signaling. On day 14, ASC/TERT1 cells showed a clear accumulation of upregulated osteogenesis- and ECM-related proteins on the right side of the volcano plot, highlighting a strong pro-osteogenic and matrix-producing phenotype at the late stage of differentiation, whereas shTLR10-ASC/TERT1 cells displayed the inverse pattern, indicative of delayed maturation. Only a small subset of proliferation-related proteins remained differentially expressed, consistent with reduced proliferative activity at this stage.

To further characterize the proteomic dynamics underlying the observed trends in the volcano plots (Figure 4A–F), a focused heatmap was generated to display the expression patterns of proteins associated with osteogenesis, proliferation, and ECM organization across days 0, 7, and 14 of differentiation (Figure 4G). Log_2_ intensities in are shown relative to corresponding mock controls for both TLR10 knockin and knockdown ASC/TERT1 cells. In TLR10-ASC/TERT1 cells, osteogenesis-associated proteins, including ALPL, FHL2, TCIRG1, and BGN, were markedly upregulated by day 7 and persisted at elevated levels by day 14, reflecting enhanced osteoblastic differentiation and matrix maturation. Several ECM and ECM–receptor interaction proteins, such as NID1, THBS1, THBS2, TGM2, MXRA7, ADAMTS1, COL8A1, ICAM1, POSTN, LAMB2, COL12A1, P3H1, LAMC1, and FBLN1, also showed progressive induction over time, consistent with the establishment of a mineralizing extracellular environment. Conversely, shTLR10-ASC/TERT1 cells exhibited the opposite expression pattern, with significantly reduced osteogenic and ECM protein levels and enhanced expression of proliferation-associated factors, such as IGFBP7, suggesting impaired matrix remodeling and delayed differentiation.

Together, these proteomic profiles illustrate that TLR10 dynamically modulates MSC differentiation. TLR10 overexpression has been demonstrated to promote osteogenic and ECM-associated protein expression over time, whereas its knockdown results in persistent proliferation-associated protein expression and hampers osteogenic maturation. These findings support a regulatory role for TLR10 in orchestrating the balance between proliferation and matrix-driven osteogenic differentiation.

### 3.4. Calcitriol Modulates TLR Signaling in ASC/TERT1 Cells Through Reciprocal Regulation of TLR10, TLR2, and TLR4

**Figure 5:**
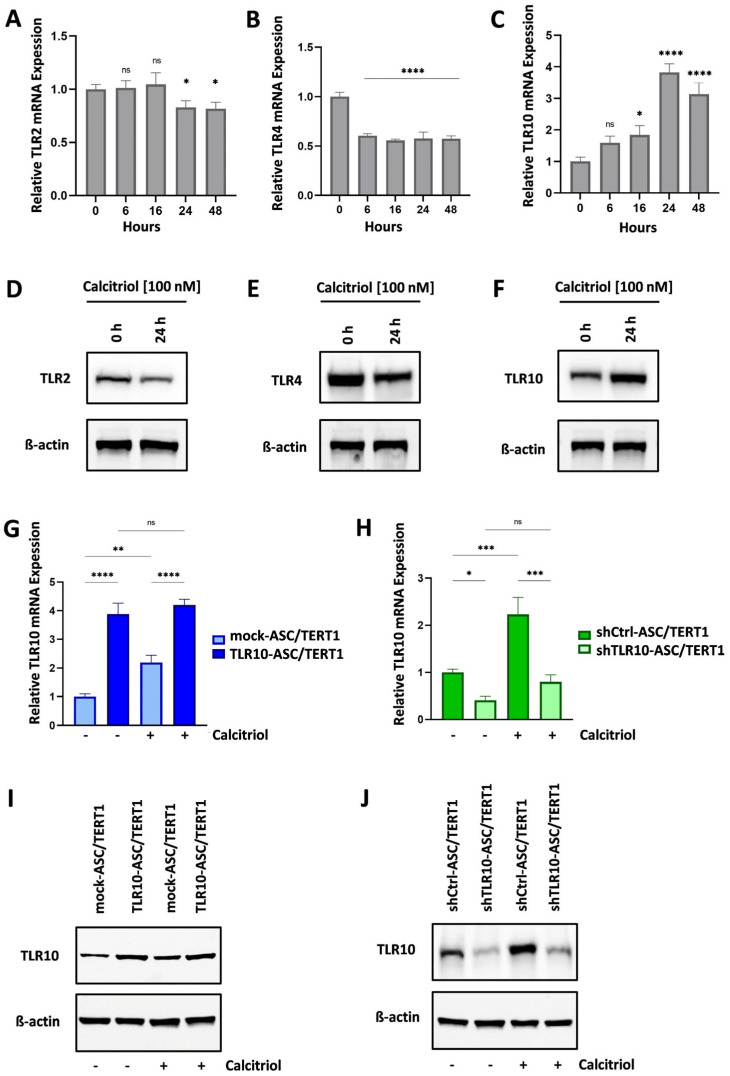
Analysis of TLR10, TLR2, and TLR4 expression in ASC/TERT1 cells following calcitriol treatment. (**A**–**C**) Quantitative PCR analysis of TLR10, TLR2, and TLR4 mRNA levels after 0–48 h of 100 nM Calcitriol treatment. Data shown as mean ± standard deviation (*n* = 3). Statistical analysis: One-way ANOVA with Dunnett’s post hoc test. ns = not significant, * *p* < 0.05, **** *p* < 0.0001. (**D**–**F**) Western blot of TLR2, TLR4, and TLR10 protein levels after 24 h of 100 nM Calcitriol treatment. β-actin served as loading control. (**G**,**H**) Quantitative PCR analysis of TLR10 mRNA levels, and (**I**,**J**) Western blot analysis of TLR10 protein levels in ASC/TERT1 cells transduced with empty vector (mock-ASC/TERT1 or shCtrl-ASC/TERT1), TLR10-expressing vector (TLR10-ASC/TERT1), or TLR10-silencing vector (shTLR10-ASC/TERT1) and exposed to 100 nM Calcitriol for 24 h or left untreated. (**G**,**H**) Data are shown as mean ± standard deviation (*n* = 3). Statistical analysis: One-way ANOVA with Šidák’s post hoc test. ns = not significant, * *p* < 0.05, ** *p* < 0.01, *** *p* < 0.001, **** *p* < 0.0001. (**I**,**J**) β-Actin was used as a loading control.

To investigate the regulatory effect of calcitriol on TLR expression in MSCs, TLR2, TLR4, and TLR10 mRNA and protein levels were analyzed following treatment of wild-type ASC/TERT1 cells with 100 nM calcitriol for 0–48 h. Quantitative RT-PCR analysis revealed a moderate decrease in TLR2 expression over time (~20%) (Figure 5A), whereas TLR4 expression declined more markedly, reaching approximately 60% of basal levels by 6 h and remaining at comparable levels up to 48 h (Figure 5B). In contrast, TLR10 mRNA levels increased significantly in a time-dependent manner, rising nearly fourfold after 24 h and threefold after 48 h of calcitriol treatment (Figure 5C). Western blot analysis following 24 h treatment with 100 nM calcitriol confirmed these transcriptomic trends: TLR2 (Figure 5D) and TLR4 (Figure 5E) protein levels decreased, whereas TLR10 protein expression was strongly induced (Figure 5F). These findings indicate that calcitriol selectively upregulates TLR10 expression while downregulating TLR2 and TLR4, suggesting a vitamin D-mediated shift toward a distinct TLR signaling profile in MSCs.

To further explore the responsiveness of TLR10 to calcitriol under conditions of altered receptor expression, we examined TLR10 mRNA and protein levels in engineered TLR10 knockin and knockdown models. In mock-transduced MSCs, calcitriol increased TLR10 mRNA levels by approximately twofold. The TLR10-ASC/TERT1 cells exhibited around fourfold higher basal TLR10 expression relative to mock cells, but calcitriol treatment did not further enhance expression, suggesting that a transcriptional plateau had been reached (Figure 5G). Consistently, Western blot analysis confirmed robust TLR10 protein abundance in TLR10-ASC/TERT1 cells before and after calcitriol administration (Figure 5I). In contrast, shTLR10-ASC/TERT1 cells displayed markedly reduced basal TLR10 expression (40–60% of control levels), and calcitriol induced only a modest increase (~30%), reflecting the reduced basal transcript levels in knockdown cells and suggesting that the transcriptional response to calcitriol is limited under conditions of TLR10 suppression (Figure 5H,J).

### 3.5. TLR10 Contributes to Calcitriol-Driven Osteogenic Differentiation in ASC/TERT1 Cells

To investigate whether TLR10 is required for the pro-osteogenic effects of vitamin D signaling, TLR10 knockdown and corresponding control cells were differentiated for 14 days in the presence or absence of 100 nM calcitriol. Cellular metabolism/proliferation were monitored using the PrestoBlue assay, while osteogenic differentiation was assessed by RT-qPCR for key marker genes (*COL1A2*) (proliferation/early matrix formation), *ALPL* (matrix maturation), and *BGLAP*/osteocalcin (mineralization)) and by Alizarin Red S staining to visualize calcium deposition.

**Figure 6:**
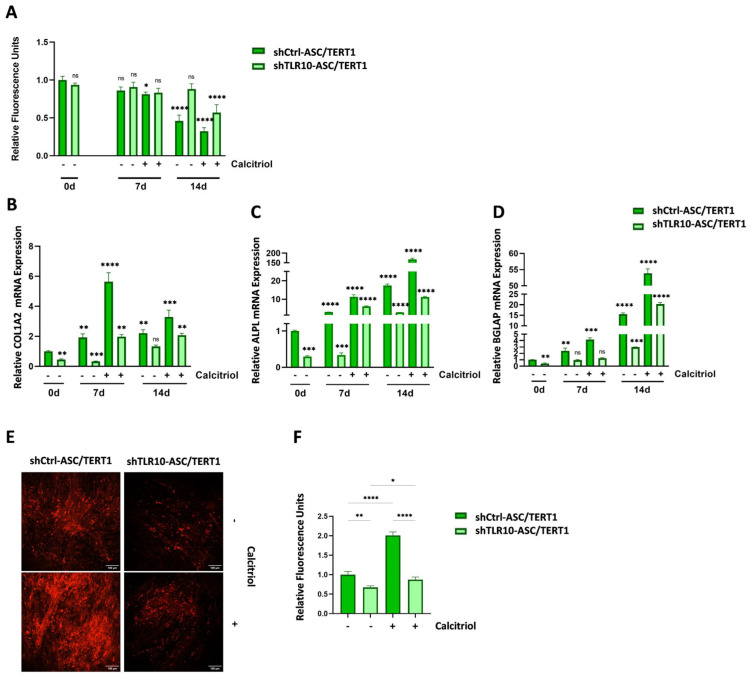
Effects of calcitriol treatment on osteogenic differentiation in TLR10-modulated ASC/TERT1 cells. ASC/TERT1 cells with TLR10 knockdown (shTLR10-ASC/TERT1) and mock controls (shCtrl-ASC/TERT1) were cultured with or without calcitriol treatment. (**A**) Cellular metabolic activity, measured by PrestoBlue at days 0, 7, and 14. (**B**–**D**) Relative mRNA expression of collagen, *ALPL*, and (**D**) osteocalcin. (**A**–**D**) Data are presented as mean ± SD (*n* = 3). Statistical analysis was performed using two-way ANOVA with Šidák’s post hoc test. ns = not significant; * *p* < 0.05; ** *p* < 0.01; *** *p* < 0.001; **** *p* < 0.0001. TLR10 knockin cells at days 0–7 were normalized to mock-ASC/TERT1 cells at day 0, and TLR10 knockdown cells were normalized to shCtrl-ASC/TERT1 cells at day 0. (**E**,**F**) Extracellular matrix mineralization, assessed by Alizarin Red S staining. (**E**) Scale bars = 100 µm. (**F**) Data are presented as mean ± SD (*n* = 3). Statistical analysis was performed using one-way ANOVA with Šidák’s post hoc test. * *p* < 0.05; ** *p* < 0.01; **** *p* < 0.0001.

At days 0 and 7, PrestoBlue measurements indicated comparable metabolic activity between control and TLR10-silenced cells, regardless of calcitriol treatment (Figure 6A). By day 14, metabolic activity declined significantly in control cells, reflecting normal differentiation-associated growth arrest, whereas shTLR10-ASC/TERT1 cells retained higher metabolic activity, consistent with delayed osteogenic progression. Notably, calcitriol supplementation partially reduced metabolic activity in shTLR10-ASC/TERT1 cells, suggesting a minor induction of differentiation in the absence of TLR10 (Figure 6A).

RT-qPCR analysis revealed that in untreated shTLR10-ASC/TERT1 cells, expression of *COL1A2*, *ALPL*, and *BGLAP* was markedly reduced compared to control across all time points, confirming impaired osteogenic gene induction. Calcitriol treatment partially restored marker expression, reaching levels approaching, but still significantly lower than, those observed in control cells, indicating that calcitriol promotes osteogenic differentiation, although this effect remains attenuated in the absence of TLR10 (Figure 6B–D).

Matrix mineralization was further evaluated by Alizarin Red S staining on day 14 to assess calcium deposition in TLR10 knockdown and control cells, with and without calcitriol (Figure 6E). Without calcitriol, the control cells displayed a characteristic network of intensely stained mineralized nodules, indicating robust calcium deposition and mature osteogenic differentiation. In contrast, shTLR10-ASC/TERT1 cells showed markedly weaker staining, with sparse and diffuse calcium deposits, confirming a pronounced impairment in matrix mineralization. Upon calcitriol supplementation, mineralization was substantially enhanced in both cell types. Control cultures exhibited denser and more extensive mineralized matrices, with uniform and intense Alizarin Red S. Notably, calcitriol-treated shTLR10-ASC/TERT1 cells also displayed augmented calcium deposition, though staining remained less intense and more heterogeneous than in treated controls (Figure 6E). Quantification of the relative fluorescence signal corroborated these observations, demonstrating a significant increase in mineralization upon calcitriol treatment, while the overall mineralization capacity in TLR10-deficient cells remained lower than in controls (Figure 6F). Together, these findings indicate that calcitriol promotes osteogenic differentiation in ASC/TERT1 cells, while TLR10 deficiency limits the magnitude of this response.

## 4. Discussion

This study provides evidence that TLR10 functions as a regulator of osteogenic differentiation and vitamin D-mediated signaling in human adipose-derived MSCs. Through complementary knockin and knockdown approaches, we demonstrate that TLR10 enhances osteogenic differentiation, ECM deposition, and calcification, whereas its silencing maintains proliferative activity and impairs osteoblast maturation. These findings expand the known functions of TLR10 beyond immune regulation [26,37] and position it as a molecular linker of innate immune and osteogenic signaling pathways in MSCs.

A notable aspect of our data is the asymmetry in proteomic responses to TLR10 modulation. Although TLR10 expression increased ~8-fold in the knockin MSC line and was reduced to approximately 40–60% of control levels in the knockdown, the latter exhibited markedly broader changes. This disproportion is consistent with TLR10′s role as a modulatory, rather than purely activating receptor within the TLR family. Structural and biochemical studies have shown that TLR10 forms both homodimers and heterodimers with TLR1, TLR2 and TLR6 [38,39], modulating MyD88- and NF-κB-dependent pathways without triggering classical inflammatory cascades [40]. Overexpression may therefore be constrained by limited dimerization partners or ligands, whereas partial depletion could destabilize receptor complexes, releasing inhibitory control over TLR1/2/6 signaling and activating compensatory stress or inflammatory pathways. Such dynamics resemble the amplification of responses observed when negative regulators in innate immune signaling, such as phosphatases or deubiquitinases, are reduced [41,42,43,44].

Quantitative proteomics revealed reciprocal effects of TLR10 overexpression and silencing on osteogenesis- and vitamin D-responsive networks. Overexpression upregulated proteins associated with ECM organization (COL12A1, PLEC, ACTN1, TNS1), metabolic adaptation (ACOX1), and mineralization (ALPL), whereas knockdown suppressed these proteins. These data support a model in which TLR10 facilitates the transition from a proliferative to a differentiating phenotype—an essential step for osteoblast maturation. Notably, several of the identified proteins directly reflect the observed functional phenotype, including increased ALPL expression, corresponding to enhanced mineralization, and upregulation of ECM-associated proteins, such as COL12A1 and PLEC, consistent with increased matrix deposition. By contrast, prior studies of TLR2 and TLR4 in bone biology primarily report inhibitory effects on osteogenesis under inflammatory conditions, highlighting TLR10′s unique differentiation-supportive role [45,46,47,48].

TLR10 appears to integrate metabolic, proliferative, and ECM-remodeling signals. Functional assays indicate that TLR10 promotes the metabolic shift characteristic of osteogenic differentiation, whereas its depletion preserves proliferation. Temporal proteomic profiling further showed that TLR10 dynamically orchestrates the balance between proliferation and differentiation, with knockin cells exhibiting upregulation of osteogenesis-associated proteins (ALPL, FHL2, TCIRG1) and ECM components (NID1, THBS1/2, COL12A1), while knockdown cells maintain proliferation-associated proteins (e.g., IGFBP7) and delayed ECM remodeling. Together, these findings indicate that TLR10 contributes to MSC fate decisions beyond its canonical immune functions.

A central discovery of this study is the reciprocal regulation between TLR10 and vitamin D signaling. Calcitriol selectively upregulates TLR10 while damping TLR2 and TLR4, shifting the TLR profile toward differentiation-supportive signaling. This finding complements prior observations that vitamin D downregulates TLR2 and TLR4 in monocytes and microglial cells to limit inflammatory activation [31,49,50]. In TLR10-deficient cells, calcitriol partially rescued osteogenic gene expression and mineralization, indicating that vitamin D promotes differentiation independently of TLR10, but achieves maximized effectiveness when TLR10 is present. These findings position TLR10 as a critical mediator linking vitamin D signaling to osteogenic commitment. The overlap between TLR10- and VDR-regulated networks, including shared induction of ALPL and MYLK, supports convergent signaling, in which TLR10 amplifies vitamin D-driven transcriptional and structural programs during osteoblast maturation.

These findings can be further contextualized within the broader framework of immunometabolic and neuroendocrine regulation of mesenchymal stem cell fate. Emerging evidence indicates that non-canonical signaling molecules, including metabolic cues and neuropeptide factors, modulate MSC differentiation and plasticity through anti-inflammatory and trophic mechanisms. Such pathways extend beyond classical osteogenic regulators and contribute to lineage commitment by integrating immune, metabolic, and microenvironmental signals [51,52]. In this context, TLR10 may represent a complementary regulatory node, linking innate immune signaling with differentiation-supportive pathways, potentially acting in concert with vitamin D-mediated and other non-canonical regulatory mechanisms.

Collectively, these findings identify TLR10 as a key regulator of osteogenesis, coordinating immune modulation, metabolic reprogramming, ECM deposition, and vitamin D-responsive pathways. Dysregulation of TLR10 may therefore contribute to pathological states characterized by impaired bone formation, including chronic inflammation, vitamin D deficiency, or osteoporosis.

Despite the strong in vitro evidence, several limitations should be acknowledged. First, the study was conducted in immortalized ASC/TERT1 MSCs, which provide a robust and reproducible model, but may not fully replicate the heterogeneity of primary MSCs or in vivo microenvironments. Validation in primary MSCs would strengthen the translational relevance of these findings. Second, the mechanisms by which TLR10 directly activate vitamin D receptor signaling remain to be elucidated. Whether TLR10 directly engages VDR-regulated transcriptional complexes or acts through intermediary signaling cascades, such as PI3K-Akt or MAPK, requires further investigation [37,53]. Third, although our proteomic analysis revealed signatures consistent with osteogenic differentiation, the endogenous ligands and downstream adapters mediating TLR10 signaling in MSCs remain unknown. Finally, in vivo validation in bone regeneration or metabolic bone disease models will be essential to confirm the physiological relevance of the TLR10–vitamin D axis.

## Data Availability

The original contributions presented in this study are included in the article/Supplementary Material. Further inquiries can be directed to the corresponding author(s).

## Supplementary Materials

The following supporting information can be downloaded at: https://www.mdpi.com/article/10.3390/cells15080697/s1. Figure S1: Representative morphology of ASC/TERT1 cultures used in this study. Figure S2: Pathway enrichment and network analysis of differentially expressed proteins (DEPs) in TLR10-overexpressing ASC/TERT1 cells. Figure S3: Cellular component enrichment and comparative analysis of differentially expressed proteins (DEPs) in TLR10-overexpressing ASC/TERT1 cells. Figure S4: Enrichment and comparative analysis of differentially expressed proteins (DEPs) in TLR10-silenced ASC/TERT1 cells. Figure S5: Biological process enrichment and comparative analysis of differentially expressed proteins (DEPs) in TLR10-overexpressing ASC/TERT1 cells. Figure S6: Biological process enrichment and comparative analysis of differentially expressed proteins (DEPs) in TLR10-silenced ASC/TERT1 cells. Figure S7: Molecular function enrichment and comparative analysis of differentially expressed proteins (DEPs) in TLR10-overexpressing ASC/TERT1 cells. Figure S8: Molecular function enrichment and comparative analysis of differentially expressed proteins (DEPs) in TLR10-silenced ASC/TERT1 cells. Table S1: Full set of proteins identified through our proteomic analysis and deposited in PRIDE.

## Abbreviations

ACN	Acetonitrile
ACOX1	Acyl-CoA oxidase 1
ADAMTS1	A disintegrin and metalloproteinase with thrombospondin motifs 1
ALPL	Alkaline phosphatase, biomineralization associated
ANOVA	Analysis of variance
ASC/TERT1	Adipose-derived mesenchymal stem cell transformed with human telomerase reverse transcriptase gene
BCA	Bicinchoninic Acid
BGLAP	Bone gamma-carboxyglutamate protein
BGN	Biglycan
CCL17	C-C motif chemokine ligand 17
cDNA	Complementary deoxyribonucleic acid
COL12A1	Collagen type XII alpha 1 chain
COL1A2	Collagen type I alpha 1 chain
DAMP	Damage-associated molecular pattern
DAVID	Database for annotation, visualization, and integrated discovery
DEP	Differentially expressed proteins
DNA	Deoxyribonucleic acid
dNTP	Deoxynucleoside triphosphate
ECL	Enhanced Chemiluminescnece
ECM	Extracellular matrix
EDTA	Ethylenediaminetetraacetic acid
EGM	Endothelial growth medium
EV	Extracellular vesicle
FBLN1	Fibulin 1
FHL2	Four and a Half LIM Domains Protein 2
H_2_O	Water
HRP	Horseradish-peroxidase
IAA	Iodoacetamide
ICAM1	Intercellular adhesion molecule 1
IGFBP7	Insulin-like growth factor-binding protein 7
IL	Interleukin
IPO8	Importin 8
KEGG	Kyoto Encyclopedia of Genes and Genomes
LAMB2	Laminin subunit beta-2
LAMC1	Laminin subunit gamma 1
LB	Luria Broth
Log	Logarithm
Lys-C	Lysyl endopeptidase
MAPK	Mitogen-activated protein kinase
MGB	Minor groove binder
mock-ASC/TERT1	Adipose-derived mesenchymal stem cells transfected with an empty vector (mock control to TLR10-ASC/TERT1)
mRNA	Messenger ribonucleic acid
MSC	Mesenchymal stem cell
MXRA1	MX Dynamin Like GTPase 1
MyD88	Myeloid differentiation primary response 88
MYLK	Myosin light chain kinase
NaCl	Sodium chloride
NF-κB	Nuclear factor kappa-light-chain-enhancer of activated B cells
NID1	Nidogen-1
nM	Nanomolar
ns	Not significant
P3H1	Prolyl 3-hydroxylase 1
PAMP	Pathogen-associated molecular pattern
PBS	Phosphate-buffered saline
PI3K/Akt	Phosphoinositide 3-kinase/Protein kinase B
PLEC	Plectin
POSTN	Periostin
PRIDE	Proteomics Identifications
PRR	Pattern recognition receptor
RFU	Relative Fluorescence Units
RT-qPCR	Reverse transcription–quantitative polymerase chain reaction
RUNX2	Runt-related transcription factor 2
RXR	Retinoid X receptor
SD	Standard deviation
SDS-PAGE	Sodium dodecyl sulfate polyacrylamide gel electrophoresis
shCtrl-ASC/TERT1	Adipose-derived mesenchymal stem cells transfected with an empty vector (mock control for shTLR10-ASC/TERT1)
shTLR10-ASC/TERT1	Adipose-derived mesenchymal stem cells stably expressing a TLR10 knockdown vector
SOC	Super optimal medium with catabolic repressor
TCEP	Tris(2-carboxyethyl)phosphine
TCIRG1	T cell immune regulator 1, ATPase H+ transporting V0 subunit a3
TGF-ß1	Transforming growth factor beta 1
TGM2	Transglutaminase 2
THBS	Thrombospondin 1
TL	Transmitted light
TLR	Toll-like receptor
TLR10-ASC/TERT1	Adipose-derived mesenchymal stem cells stably expressing a TLR10 knockin vector
TNF-α	Tumor necrosis factor alpha
TNS1	Tensin 1
TRIF	TIR-domain-containing adapter-inducing interferon-beta
Tris-HCl	Tris(hydroxymethyl)aminomethane hydrochloride
VDR	Vitamin D receptor
