# Supplementary material for: The TLR10–Vitamin D Axis Facilitates Osteogenic Differentiation of Mesenchymal Stem Cells In Vitro
Source: Cells. 2026 Apr 15;15(8):697. doi: 10.3390/cells15080697 (PMC13115012; doi:10.3390/cells15080697)
Supplement: Supplementary file 1 [file cells-15-00697-s001.zip › Supplementary material 1.pdf]

Figure S1

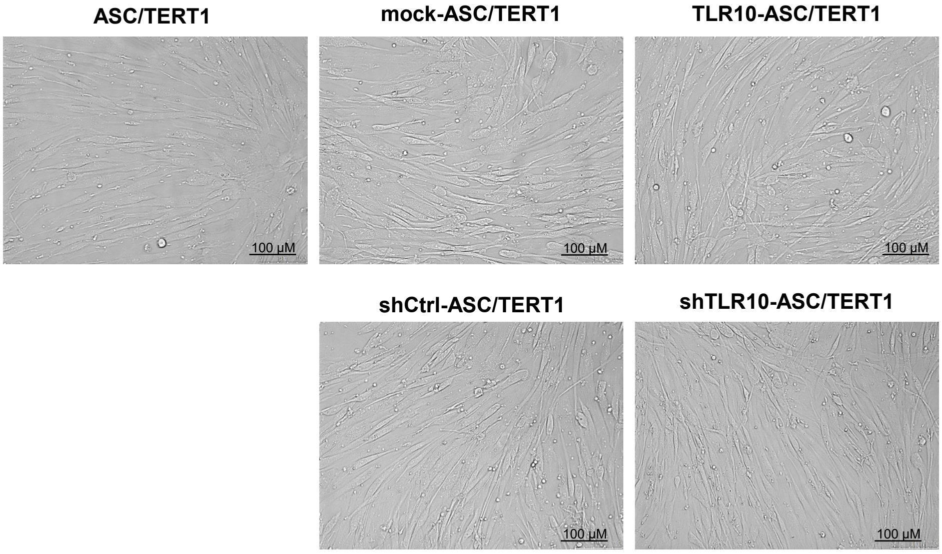

Figure S1: Representative morphology of the ASC/TERT1 cultures used in this study. Phase-contrast microscopy images are shown for the following: wild-type ASC/TERT1; mock-transfected controls (mock-ASC/TERT1); TLR10-knockin cells (TLR10-ASC/TERT1); control shRNA cells (shCtrl-ASC/TERT1); and TLR10 knockdown cells (shTLR10-ASC/TERT1). No overt morphological differences were observed between the control, knock-in, or knockdown conditions under standard culture conditions. Scale bars = 100 μm.

Figure S2

A Pathways enriched from proteins upregulated in TLR10-ASC/TERT1

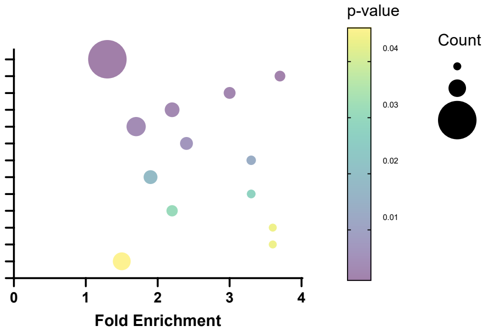

B Overlap of metabolic pathways enriched among proteins upregulated in TLR10-ASC/TERT1 cells and associated with vitamin D signaling and osteogenesis

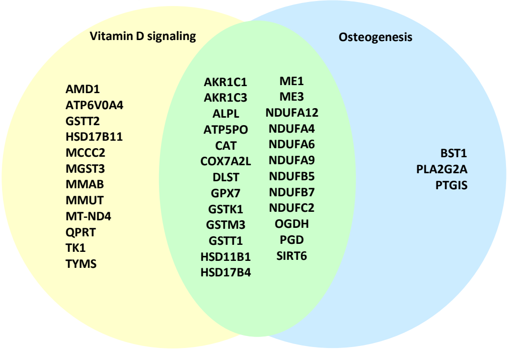

C Network analysis of pathways enriched among proteins upregulated in TLR10-ASC/TERT1 cells and associated with vitamin D signaling and osteogenesis

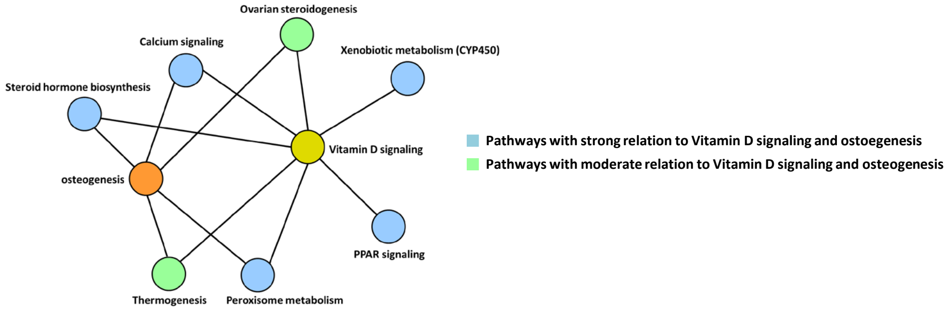

D Pathways enriched from proteins downregulated in TLR10-ASC/TERT1

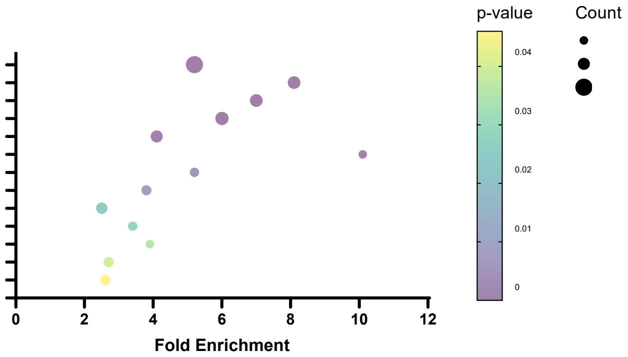

Figure S2: Pathway enrichment and network analysis of differentially expressed proteins (DEPs) in TLR10-overexpressing ASC/TERT1 cells. (A, D) Bubble plots of KEGG pathway enrichment (DAVID) for significantly ($p < 0.05$) upregulated **(A)** and downregulated **(D)** proteins in TLR10-overexpressing ASC/TERT1 cells versus mock controls; bubble size indicates the number of DEPs, color intensity (violet → yellow) reflects the p-value, and the x-axis shows fold enrichment. **(B)** Venn diagram illustrating shared and unique DEPs related to osteogenesis and vitamin D signaling. **(C)** Network analysis based on KEGG enrichment among upregulated proteins reveals an interconnected cluster of pathways associated with osteogenic differentiation and vitamin D–mediated signaling.

Figure S3

A Cellular components enriched from proteins upregulated in TLR10-ASC/TERT1

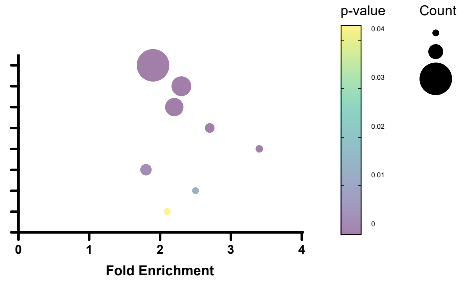

B Overlap of cellular components enriched among proteins upregulated in TLR10-ASC/TERT1 cells and associated with vitamin D signaling and osteogenesis

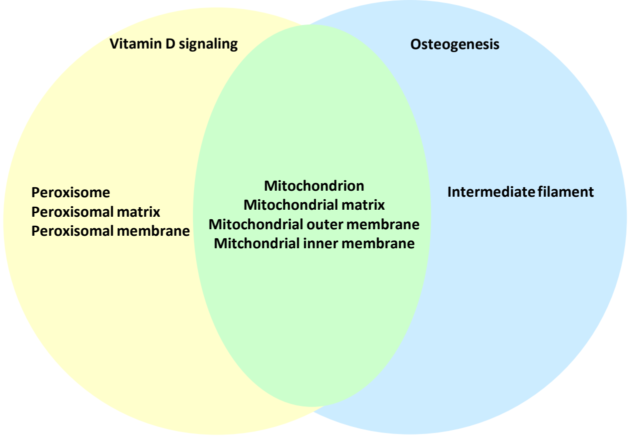

C Cellular components enriched from proteins downregulated in TLR10-ASC/TERT1

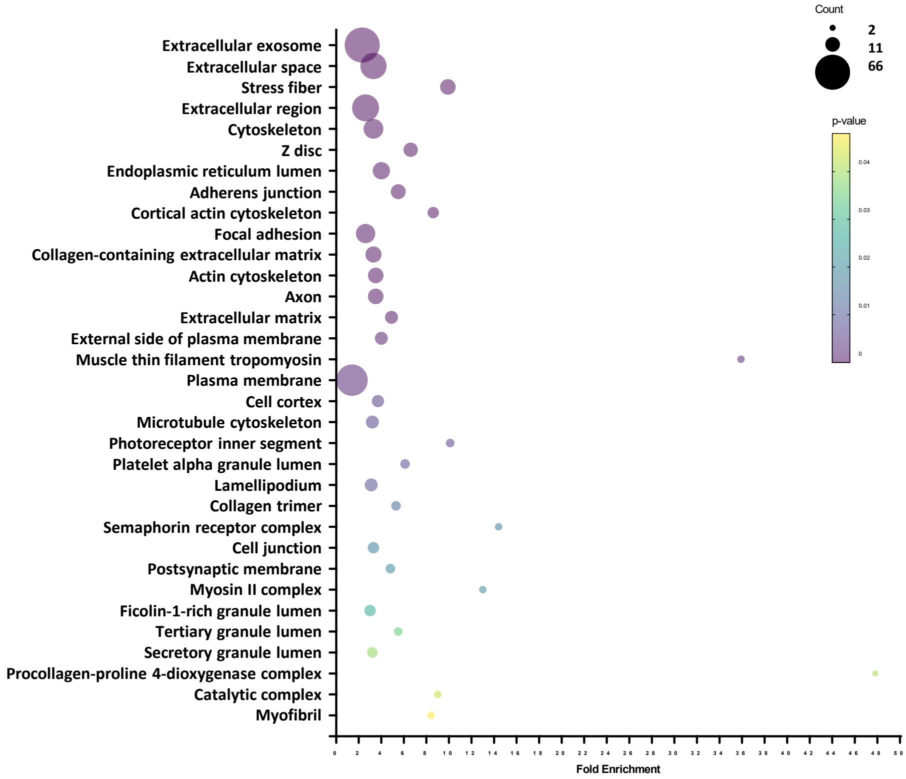

Figure S3: Cellular component enrichment and comparative analysis of differentially expressed proteins (DEPs) in TLR10-overexpressing ASC/TERT1 cells. (A, C) Bubble plots showing Gene Ontology (GO) cellular component enrichment, determined via DAVID, for significantly ($p < 0.05$) upregulated **(A)** and downregulated **(C)** proteins in TLR10-overexpressing ASC/TERT1 cells relative to mock controls; bubble size represents the number of DEPs, color intensity (violet → yellow) denotes p-value significance, and the x-axis indicates fold enrichment. **(B)** Venn diagram illustrating shared and unique DEPs related to osteogenesis and vitamin D signaling.

Figure S4

A Cellular components enriched from proteins upregulated in shTLR10-ASC/TERT1

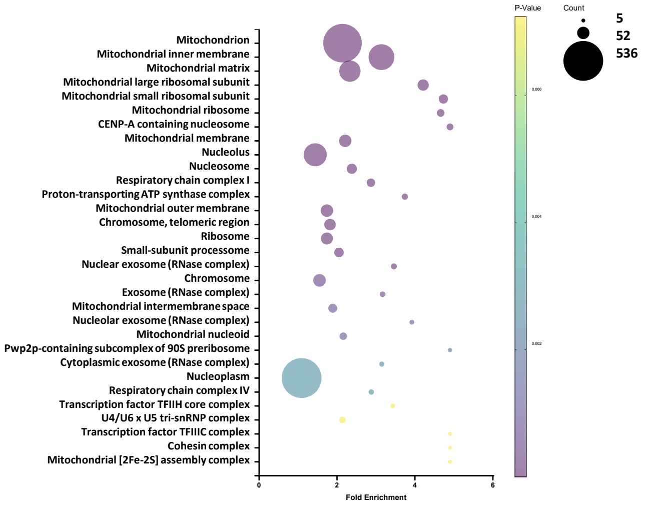

B Cellular components enriched from proteins downregulated in shTLR10-ASC/TERT1

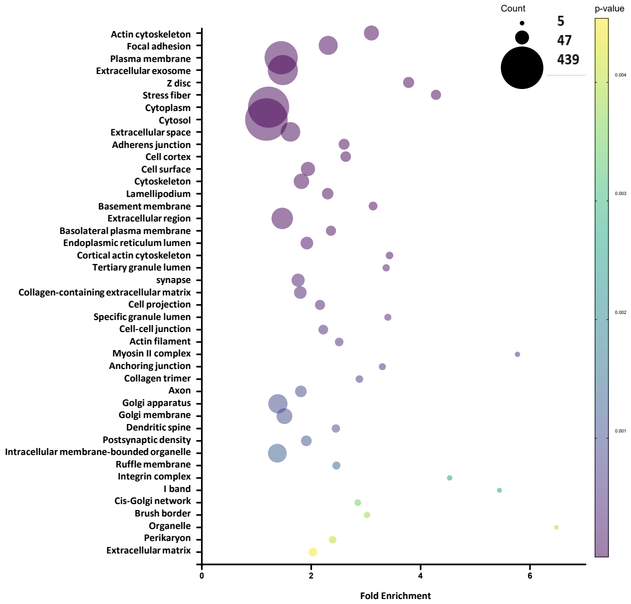

C Overlap of cellular components enriched among proteins downregulated in shTLR10-ASC/TERT1 cells and associated with vitamin D signaling and osteogenesis

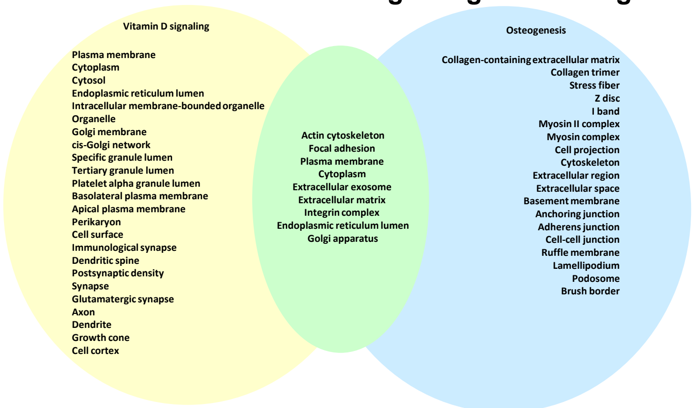

Figure S4: Enrichment and comparative analysis of differentially expressed proteins (DEPs) in TLR10-silenced ASC/TERT1 cells. (A, B) Bubble plots showing Gene Ontology (GO) cellular component enrichment, determined via DAVID, for significantly ($p < 0.05$) upregulated **(A)** and downregulated **(B)** proteins in TLR10-silenced ASC/TERT1 cells relative to mock controls; bubble size represents the number of DEPs, color intensity (violet → yellow) denotes p-value significance, and the x-axis indicates fold enrichment. **(C)** Venn diagram illustrating shared and unique DEPs related to osteogenesis and vitamin D signaling.

Figure S5

A Biological processes enriched from genes upregulated in TLR10-ASC/TERT1

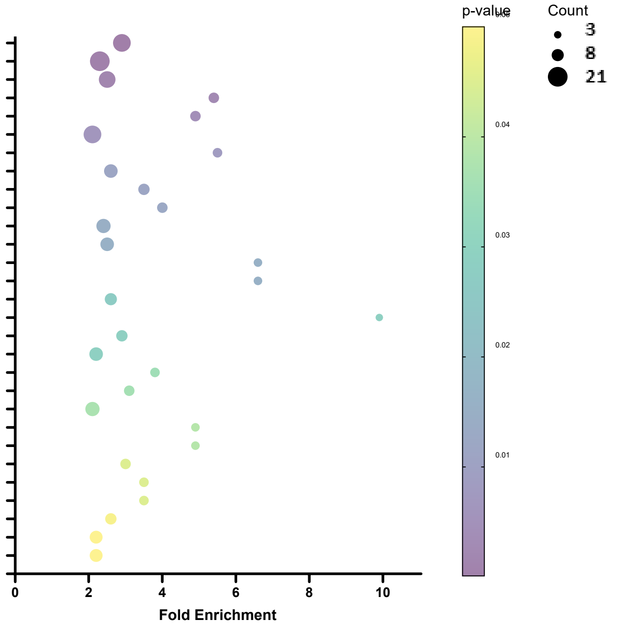

B Overlap of biological processes enriched among genes upregulated in TLR10-ASC/TERT1 cells and associated with vitamin D signaling and osteogenesis

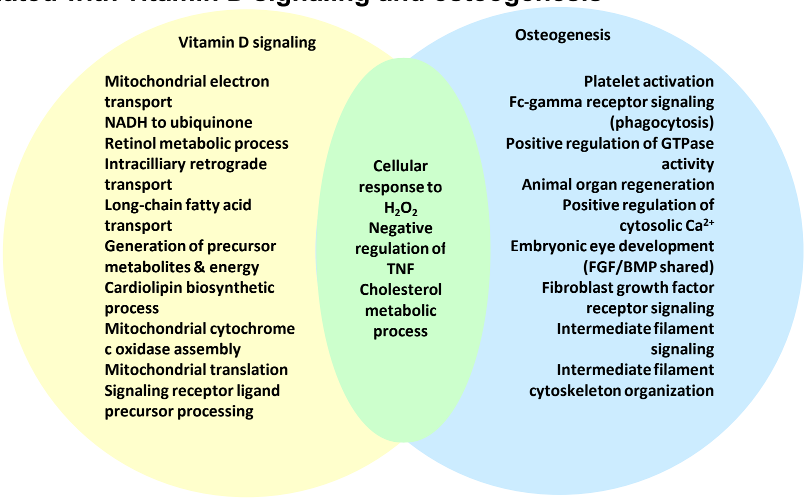

C Biological processes enriched from genes downregulated in TLR10-ASC/TERT1

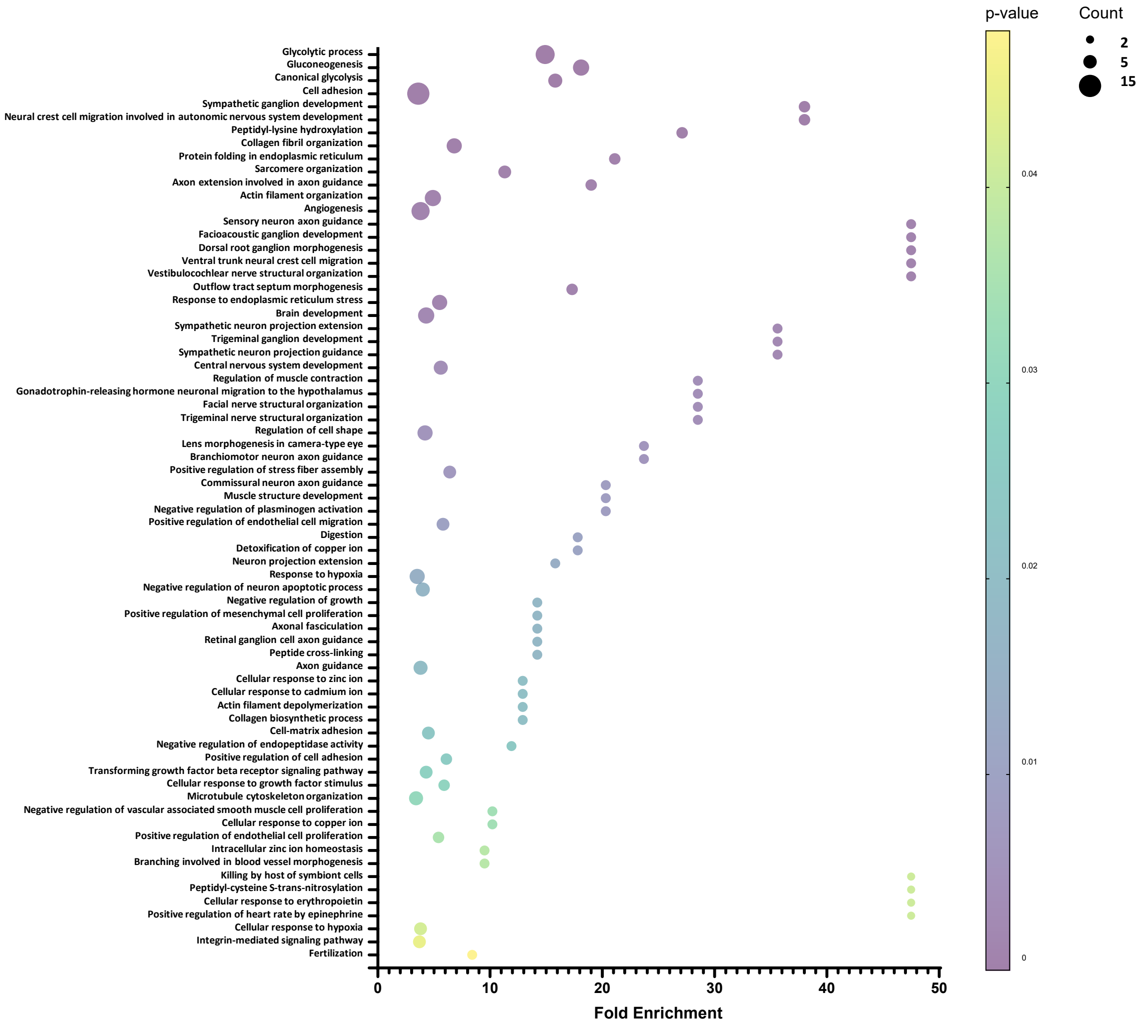

Figure S5: Biological process enrichment and comparative analysis of differentially expressed proteins (DEPs) in TLR10-overexpressing ASC/TERT1 cells. (A, C) Bubble plots showing Gene Ontology (GO) biological process enrichment, determined via DAVID, for significantly ($p < 0.05$) upregulated **(A)** and downregulated **(C)** proteins in TLR10-overexpressing ASC/TERT1 cells relative to mock controls; bubble size represents the number of DEPs, color intensity (violet → yellow) denotes p-value significance, and the x-axis indicates fold enrichment. **(B)** Venn diagram illustrating shared and unique DEPs related to osteogenesis and vitamin D signaling.

Figure S6

A Biological processes enriched from genes upregulated in shTLR10-ASC/TERT1

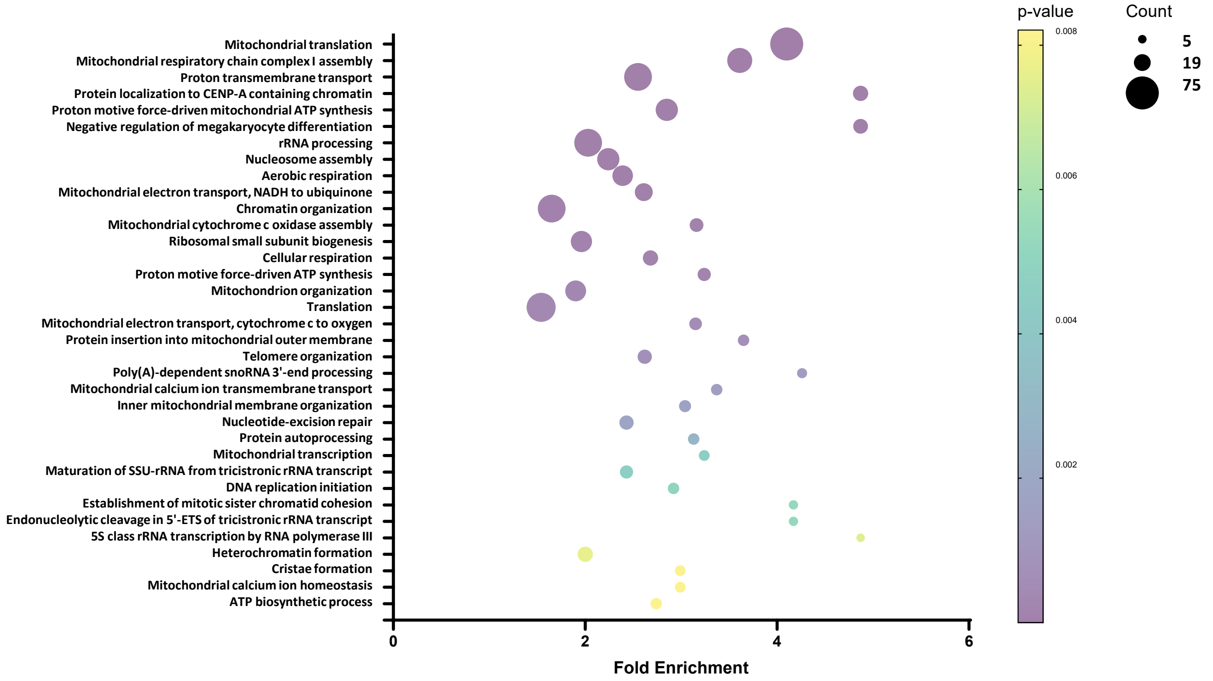

B Biological processes enriched from genes downregulated in shTLR10-ASC/TERT1

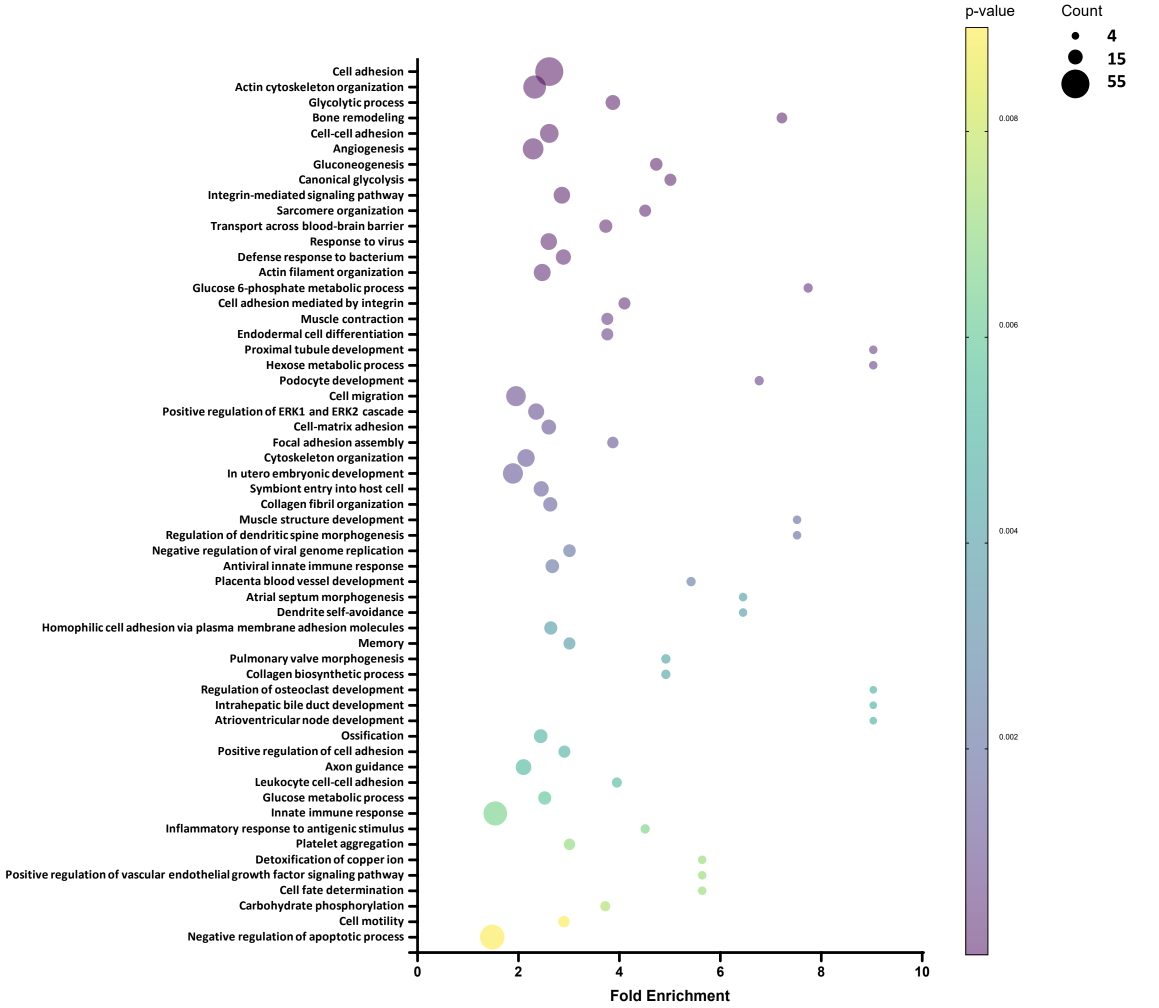

C Overlap of biological processes enriched among genes downregulated in shTLR10-ASC/TERT1 cells and associated with vitamin D signaling and osteogenesis

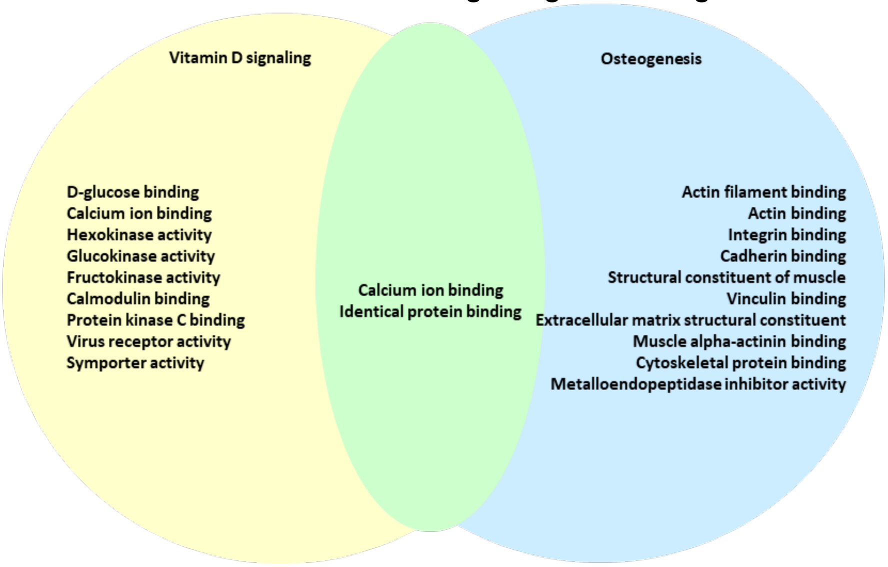

Figure S6: Biological process enrichment and comparative analysis of differentially expressed proteins (DEPs) in TLR10-silenced ASC/TERT1 cells. (A, B) Bubble plots showing Gene Ontology (GO) biological process enrichment, determined via DAVID, for significantly ($p < 0.05$) upregulated **(A)** and downregulated **(B)** proteins in TLR10-silenced ASC/TERT1 cells relative to mock controls; bubble size represents the number of DEPs, color intensity (violet → yellow) denotes p-value significance, and the x-axis indicates fold enrichment. **(C)** Venn diagram illustrating shared and unique DEPs related to osteogenesis and vitamin D signaling.

Figure S7

A Molecular function enriched from genes upregulated in TLR10-ASC/TERT1

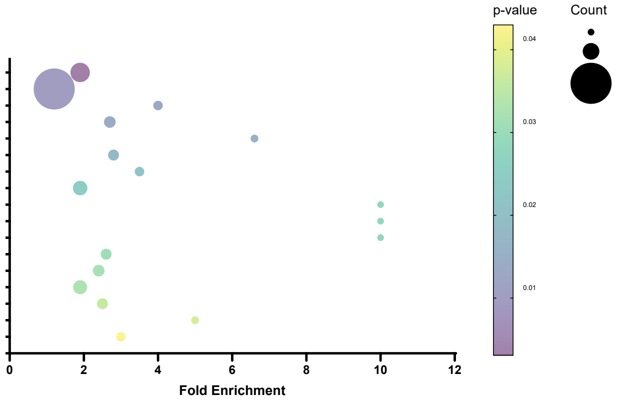

B Overlap of molecular functions enriched among genes upregulated in TLR10-ASC/TERT1 cells and associated with vitamin D signaling and osteogenesis

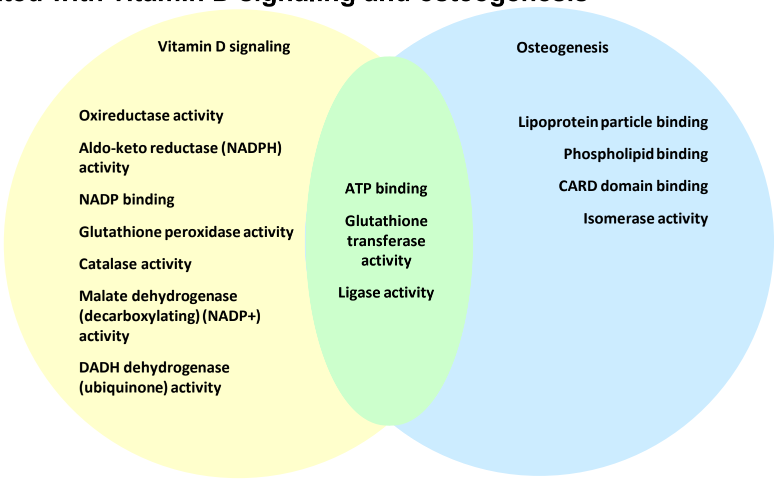

C Molecular function enriched from genes downregulated in TLR10-ASC/TERT1

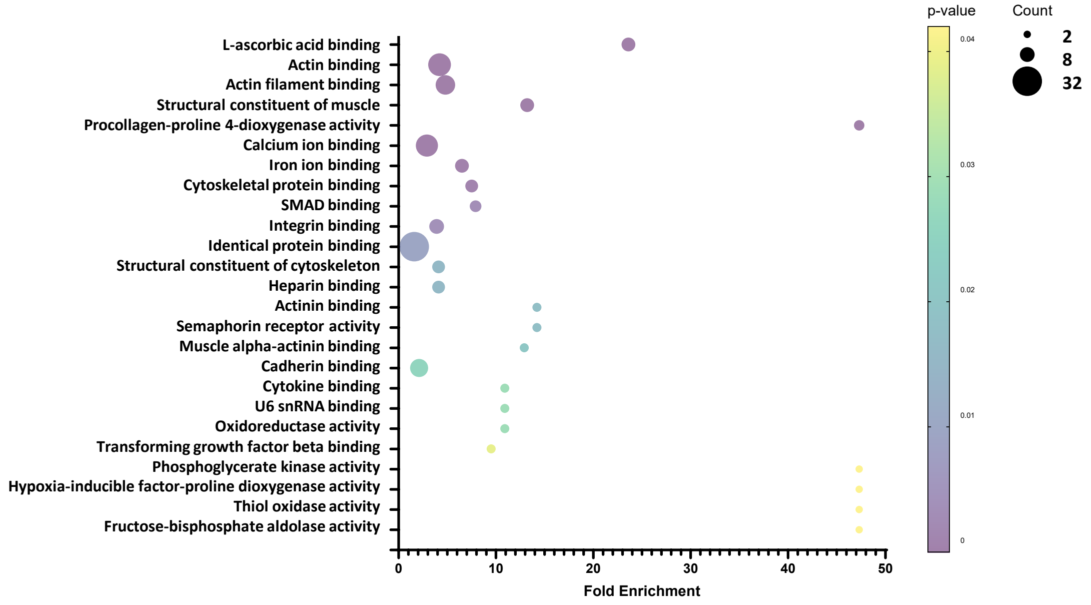

Figure S7: Molecular function enrichment and comparative analysis of differentially expressed proteins (DEPs) in TLR10-overexpressing ASC/TERT1 cells. (A, C) Bubble plots showing Gene Ontology (GO) molecular function enrichment, determined via DAVID, for significantly ($p < 0.05$) upregulated **(A)** and downregulated **(C)** proteins in TLR10-overexpressing ASC/TERT1 cells relative to mock controls; bubble size represents the number of DEPs, color intensity (violet → yellow) denotes p-value significance, and the x-axis indicates fold enrichment. **(B)** Venn diagram illustrating shared and unique DEPs related to osteogenesis and vitamin D signaling.

Figure S8

A Molecular function enriched from genes upregulated in shTLR10-ASC/TERT1

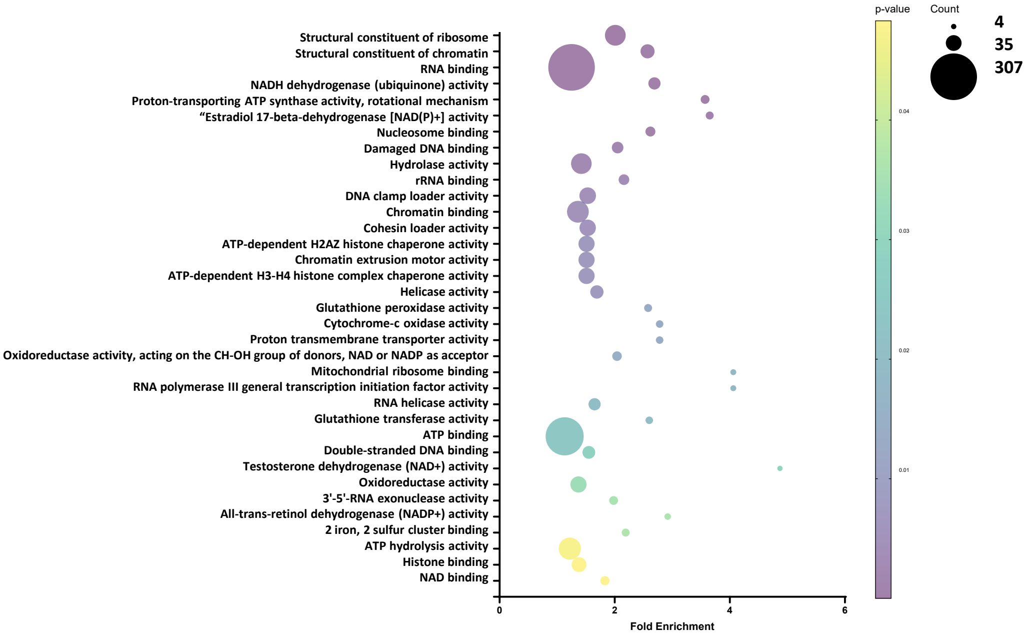

B Molecular function enriched from genes downregulated in shTLR10-ASC/TERT1

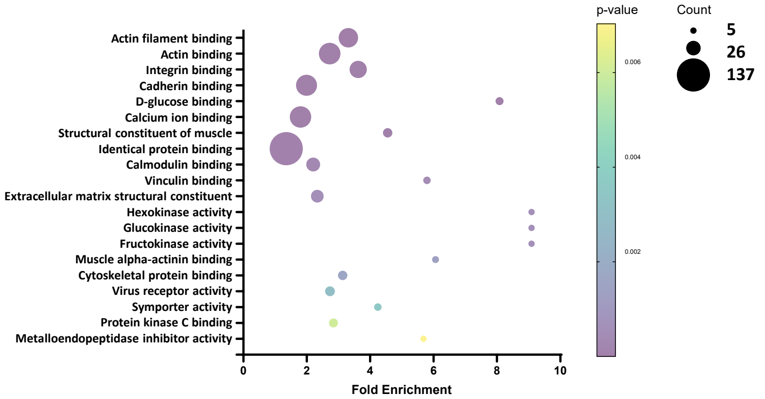

C Overlap of molecular functions enriched among genes downregulated in shTLR10-ASC/TERT1 cells and associated with vitamin D signaling and osteogenesis

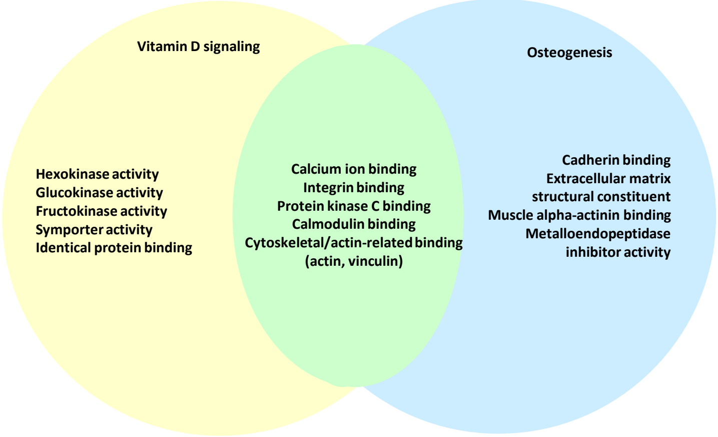

Figure S8: Molecular function enrichment and comparative analysis of differentially expressed proteins (DEPs) in TLR10-silenced ASC/TERT1 cells. (A, B) Bubble plots showing Gene Ontology (GO) molecular function enrichment, determined via DAVID, for significantly ($p < 0.05$) upregulated **(A)** and downregulated **(B)** proteins in TLR10-silenced ASC/TERT1 cells relative to mock controls; bubble size represents the number of DEPs, color intensity (violet → yellow) denotes p-value significance, and the x-axis indicates fold enrichment. **(C)** Venn diagram illustrating shared and unique DEPs related to osteogenesis and vitamin D signaling.
